# Membrane tension induces F-actin reorganization and flow in a biomimetic model cortex

**DOI:** 10.1038/s42003-023-04684-7

**Published:** 2023-03-27

**Authors:** Ryota Sakamoto, Deb Sankar Banerjee, Vikrant Yadav, Sheng Chen, Margaret L. Gardel, Cecile Sykes, Shiladitya Banerjee, Michael P. Murrell

**Affiliations:** 1 Department of Biomedical Engineering, Yale University, 10 Hillhouse Avenue, New Haven, CT USA; 2 Systems Biology Institute, 850 West Campus Drive, West Haven, CT USA; 3 Department of Physics, Carnegie Mellon University, Pittsburgh, PA 15213 USA; 4 Department of Physics, University of Chicago, Chicago, IL 60637 USA; 5 James Franck Institute, University of Chicago, Chicago, IL 60637 USA; 6 Institute for Biophysical Sciences and Pritzker School of Molecular Engineering, University of Chicago, Chicago, IL 60637 USA; 7 Laboratoire de Physique, l’Ecole Normale Supérieure, Paris, France; 8 Department of Physics, Yale University, 217 Prospect Street, New Haven, CT USA

**Keywords:** Permeation and transport, Membrane biophysics, Biophysics

## Abstract

The accumulation and transmission of mechanical stresses in the cell cortex and membrane determines the mechanics of cell shape and coordinates essential physical behaviors, from cell polarization to cell migration. However, the extent that the membrane and cytoskeleton each contribute to the transmission of mechanical stresses to coordinate diverse behaviors is unclear. Here, we reconstitute a minimal model of the actomyosin cortex within liposomes that adheres, spreads and ultimately ruptures on a surface. During spreading, accumulated adhesion-induced (passive) stresses within the membrane drive changes in the spatial assembly of actin. By contrast, during rupture, accumulated myosin-induced (active) stresses within the cortex determine the rate of pore opening. Thus, in the same system, devoid of biochemical regulation, the membrane and cortex can each play a passive or active role in the generation and transmission of mechanical stress, and their relative roles drive diverse biomimetic physical behaviors.

## Abstract (web summary)

Visualization of F-actin within liposomes during their adhesion, spreading and rupture reveals mechanical interactions of membrane and cytoskeleton can lead to complex cellular assembly in absence of biochemical regulation.

## Introduction

The surface tension of living cells determines cell shape during essential processes, including adhesion, migration, and division. Tensile stresses within the cell surface are diverse and originate within the cytoskeleton and the plasma membrane. The coordination between these stresses determines the mechanical properties of the cell^1,2^.

The activity of myosin II molecular motors generates contractile stresses by pulling on F-actin that lies beneath the plasma membrane^3,4^. Termed cortical tension, the accumulation of mechanical stresses is controlled by myosin content and activity, as well as F-actin network properties, such as cortical thickness^5^ and filament length^6^. By contrast, membrane tension may be generated by regulation of membrane surface area (endocytosis), adhesion to the extracellular matrix, or regulation of internal pressure^7^. However, as the two are physically linked, changes to cortical tension impacts membrane tension and vice versa^8,9^. For example, the onset of adhesion to the extracellular matrix elevates membrane tension, which limits the rate of cell spreading^10^ by halting the assembly of F-actin within the lamellipodia^11^. By contrast, decreased cell-scale membrane tension increases global F-actin assembly^9^. Separately, actomyosin contractility increases membrane tension through the transmission of mechanical stress through ERM-based linkages^5,12^. Thus, while there is a clear association between membrane tension and cortical tension, the internal biochemical regulation of cytoskeletal and membrane content and activity obscures an understanding of the mechanistic relationship between the two.

Giant Unilamellar Vesicles (GUVs, liposomes) are widely used to reconstitute minimal models of the cell membrane^13,14^ and cytoskeleton^15–21^. Devoid of internal biomechanical regulation, the mechanical response of liposomes is probed independent of mounted cellular response. For example, a cortical actin layer encapsulated within the liposome alters the membrane tension of the liposome^22^. Further, the actin layer determines the strength of liposome adhesion to a surface^23^ and mediates the dynamics of liposome spreading^24^. However, liposome adhesion and spreading builds membrane tension and generates strong contractile forces^25^. How these forces in turn, alter the dynamics and mechanics of the actin cortical layer are unclear. Nor is it clear how active stresses within the cortical layer influence the mechanics and dynamics of the membrane. Encapsulating the actomyosin cytoskeleton inside liposomes may allow for an understanding of how membrane tension impacts cytoskeletal organization and dynamics and vice versa.

Here, we explore the relationship between membrane tension on the organization and dynamics of the internal, actomyosin cytoskeleton. To do so, we visualize the dynamics of the cytoskeleton encapsulated within liposomes during the adhesion, spreading and rupture of the liposomes over time. During adhesion and spreading, passive stresses dominate the mechanical response. At early times, the accumulation of adhesion-induced membrane tension dramatically reorganizes the F-actin cytoskeleton, yielding cell-like F-actin structures. By contrast, at later times upon lysis, pore expansion depends upon active stresses within the actin layer. These results illustrate that basic mechanical interactions, devoid of cellular regulation can yield complex cellular assembly and behaviors.

## Results

### Liposome adhesion accumulates high membrane tension

**Fig. 1:**
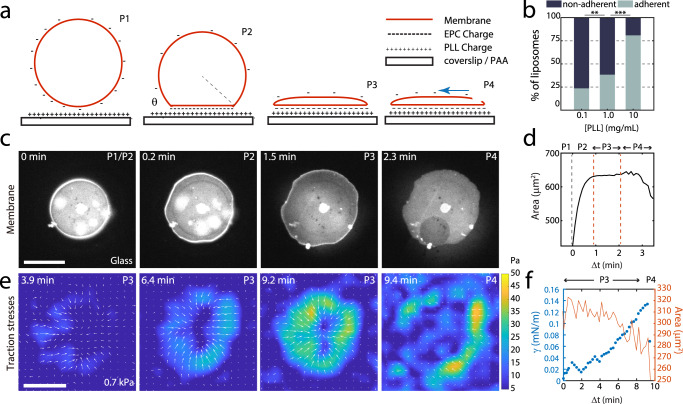
Liposome adhesion accumulates large membrane surface stresses. **a** Schematic of a spreading liposome, where adhesion is mediated by charge in the membrane and a PLL-coated surface. The surface may be glass (as in (**c**)) or a polyacrylamide gel (as in (**e**)). **b** Percentage of empty liposomes that have adhered (gray) and that have not adhered (black) to coverslips under different surface coating of PLL (0.1 mg/ml PLL, *N* = 224; 1 mg/ml PLL, *N* = 95; 10 mg/ml PLL, *N* = 210; *n* = 1 independent experiment for each condition). **c** Fluorescent lipid (OG-DHPE) in an empty liposome spreading at 10 mg/mL PLL on glass. **d** Area over time of liposome spreading in (**c**). Different phases of spreading are indicated. Time is from first observation of the liposome ($\Delta t$), within minutes of attaching to surface. **e** Traction stresses for a liposome spreading on 0.7 kPa PLL-coated gel, as measured by Traction Force Microscopy (TFM). White arrows indicate direction of stresses, and the color bar indicates the magnitude of the stress (Pa). **f** Spread area (orange) and surface tension (blue), as calculated by TFM. Scale bars are 10 μm. ** and *** stand for *p* < 0.01 and 0.001, respectively.

Empty (bare) liposomes (‘BL’, ~5–50 μm) are composed of phospholipid (Egg PC), cholesterol, DOGS-NTA-Ni, and fluorescent lipid (Oregon Green DHPE or Texas Red DHPE). They then adhere and spread onto poly-L-lysine (PLL) coated coverslips and PLL-coated polyacrylamide gels (PAA). As the liposome contacts the surface, the interaction between the negative charges in the liposome and the positively charged PLL mediates strong adhesion (Fig. 1a). A larger fraction of liposomes adheres and spreads as the PLL concentration increases (Fig. 1b). Subsequently, the liposomes spread in four phases that we denote as P1-P4 (Fig. 1c, d, Supplementary Movie 1). Upon contact, the contact area increases quickly (P1). For high PLL concentration (>1 mg/mL), this stage is rapid (<1 s) and is not captured, but has been reported elsewhere^24^. This rapid spreading is in contrast to the slower initial stages of cell adhesion and isotropic cell spreading, which occurs on the order of 100 s^26^. Subsequently, spreading continues, and contact area increases, but at a decreased rate compared to P1 (P2). When spreading on glass, there may also be an extended phase in which the liposome contact area increases minimally (P3). By contrast, when spreading on 0.7 kPa PAA, there is a nominal decrease in contact area (~10%) in P3 representing a contraction of the gel (Fig. 1e, f). On either substrate, the liposome may flatten to a ‘pancake’ shape towards the end of P3 (Supplementary Fig. 1, Supplementary Movie 2). Ultimately, a pore appears on the upper surface of liposome, and the liposome ruptures near the contact line (P4)^27^. Henceforth, we will focus on the slow dynamics of spreading (P3) and rupture of the liposome (P4).

By Traction Force Microscopy (TFM), we measure a two-dimensional accumulation of traction stress ($\vec{\sigma }$), localized principally at the boundary of the bare liposome, as has been observed previously (Fig. 1e)^25^. Here, the membrane tension (γ) is calculated from the increase in $\vec{\sigma }$ at the contact line such that the mean peripheral stress is multiplied by the perimeter (Fig. 1e, f). We further show that the tension increases during P3, terminating upon reaching the lysis tension at which point the liposome ruptures (P4). A lower bound for the lysis tension is 0.14 mN/m (as tension changes quickly in time), comparable to 0.3-5.0 mN/m as measured by other methods^24,27–30^. Having established that the spreading of liposomes increases the membrane tension in a time-dependent manner, we next explore the impact of tension on the dynamics and assembly of actomyosin encapsulated within the liposome.

### Assembly of an actomyosin cortex

**Fig. 2:**
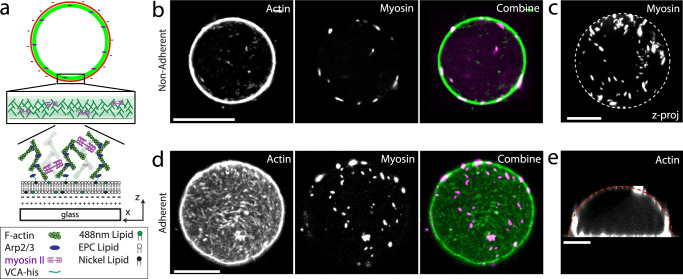
Assembly of an actomyosin cortex within a liposome. **a** Schematic of an actomyosin-encapsulated liposome. A branched actin network is nucleated from Arp2/3 locally activated by His-VCA recruited beneath the lipid bilayer membrane in the presence of nickel lipid. **b** Actin, skeletal muscle myosin II, actin and myosin combine within a non-adherent liposome (left to right). **c** A z-projection of skeletal muscle myosin II within a liposome. **d** Actin, myosin and color combine within an adherent liposome with the basal level as the focal plane. **e** Side view of a confocal z-stack of actin within a liposome encapsulating cortical actomyosin. Scale bars are 10 μm.

We then encapsulate purified G-actin and associated proteins within the liposome using the inverted emulsion technique^31^ (Fig. 2a, b). Briefly, a branched actin network is nucleated from VCA, a WASP fragment that recruits the Arp2/3 complex at the inner leaflet of the bilayer, creating a thin, but dense branched F-actin layer mimicking the cell cortex^15,32^ (“Methods”, Supplementary Note 1). The actin network density is high due to the severing and capping by cofilin and gelsolin (Supplementary Fig. 2). Moreover, skeletal myosin II dimers can be incorporated in the encapsulation mixture in the presence of Blebbistatin, an inhibitor of ATPase activity. Myosin filaments assemble and reach approximately 1–2 μm in length and are distributed uniformly across the inner surface of the liposome (Fig. 2c and Supplementary Fig. 2). Henceforth, liposomes that encapsulate actin, actin binding proteins and myosin are called actomyosin liposomes (“AML”), and those with actin but without myosin are called actin liposomes (“AL”). Emulsions are prepared at 4 °C, with a negative osmotic pressure difference (~20–40 mOsm, see “Methods”) between encapsulated and outer buffer. Liposomes are added at room temperature to coverslips coupled with high concentrations (0.1–10 mg/mL) of PLL, subsequently spreads on the PLL-coated glass surface (Fig. 2d, e). The actomyosin liposomes are illuminated with 488 nm light to deactivate Blebbistatin^33–35^, prior to each illumination with separate wavelengths to visualize the actin, myosin or membrane components.

In the absence of adhesion, myosin activity can reorganize the actin cytoskeleton (Supplementary Fig. 3) and induce shape transformations, dependent upon the density of myosin thick filaments (Supplementary Figs. 4 and 5, Supplementary Movies 3 and 4)^16^. Arp2/3 networks contract dependent upon the extent of branching^36–38^. However, as this general phenomenology has been observed previously^16,39,40^, we instead focus on the mechanics of spreading of liposomes that encapsulate cytoskeletal protein.

### Membrane adhesion localizes basal actin polymerization and depolymerization

**Fig. 3:**
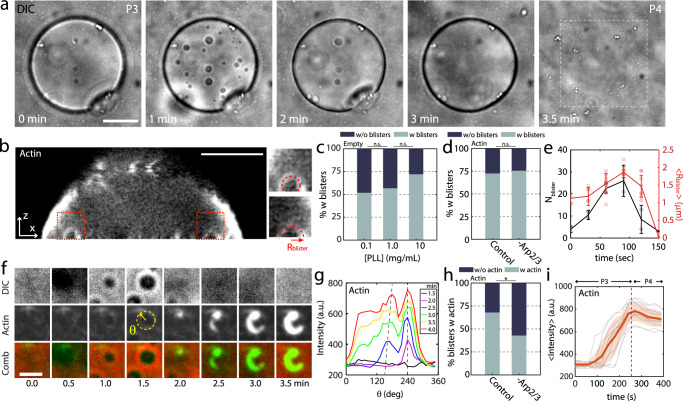
Membrane protrusions localize basal F-actin assembly. **a** Brightfield image of liposome adhesion over time on a coverslip coated with 2 mg/mL PLL. Scale bar, 10 µm. **b** Sideview of a confocal stack of F-actin within an adherent liposome. Scale bar, 10 µm. **c** Percentage of empty liposomes that have blisters (gray) and that do not (black) (0.1 mg/ml PLL, *N* = 54; 1 mg/ml PLL, *N* = 37; 10 mg/ml PLL, *N* = 171; independent experiment, *n* = 2 for each condition). **d** Percentage of actin liposomes that have blisters (gray) and that do not (black) with and without Arp2/3 at 10 mg/ml PLL (Control, *N* = 73; -Arp2/3, *N* = 37; *n* = 2 for each condition). **e** Size and number of blisters within liposomes over time. **f** Brightfield, actin and overlay of blister appearance and actin growth. Scale bar, 2 µm. **g** Angular distribution of actin fluorescence around a blister, over time. Yellow dashed circle in (**f**) indicates the region of line scan. **h** Percentage of actin liposomes that have actin enrichment in blisters (gray) and that do not (black) with and without Arp2/3 at 10 mg/ml PLL (Control, *N* = 53; -Arp2/3, *N* = 28; *n* = 2 for each condition). **i** Fluorescence over time for actin clusters (*N*_tot_ = 12). Error bars are mean ± standard deviation. * stands for *p*  <  0.05. n.s., not significant.

During P3, circular, optically dense regions on the bottom adhesive surface of the liposome are observed in transmitted light (Fig. 3a, b, Supplementary Fig. 6, Supplementary Movies 5 and 6). These features resemble phenomena previously observed in multicomponent membranes in a high ionic strength solution, described as blisters^14,41^. Briefly, blisters reflect de-adhered hemispherical protrusions into the liposome from the basal membrane surface and are associated with changes in phase behavior of the membrane^42^. The accumulation of counter charge has been shown to alter the osmotic pressure, leading to a hemispherical protrusion into the liposome. However, not all liposomes have blisters (Fig. 3c, d, 73% BL, *N* = 171; 73% AL, *N* = 73 at 10 mg/ml PLL). For those that do, they grow in size and number in early P3, and decrease in late P3, immediately prior to rupture (Fig. 3e). Concomitant with the appearance and disappearance of blisters, we observe significant changes in the intensity, spatial distribution, and the dynamics of actin fluorescence.

First, we observe an increase in actin fluorescence intensity on the basal surface, localized to the periphery of the blisters, what we term “clusters” (Fig. 3f, Supplementary Movies 5 and 6). Actin can concentrate around the perimeter of blisters in a wave-like fashion, beginning as a small point-like region that grows to encompass the circumference of the blisters (Fig. 3f, g). Wave-like assembly is consistent with Arp2/3 polymerization in vivo, where the reported time scales associated with polymerization is of the order of ~10–100 s^43–45^. In the absence of Arp2/3, cofilin or gelsolin, when blisters are present, the fraction of blisters that have associated clusters decreased (Fig. 3h). Upon the disappearance of the blisters, actin fluorescence remains enhanced prior to previous levels (Fig. 3i). The clusters of actin intensity remain after the bursting of the liposomes and are used to identify the past presence of blisters (Supplementary Fig. 7).

**Fig. 4:**
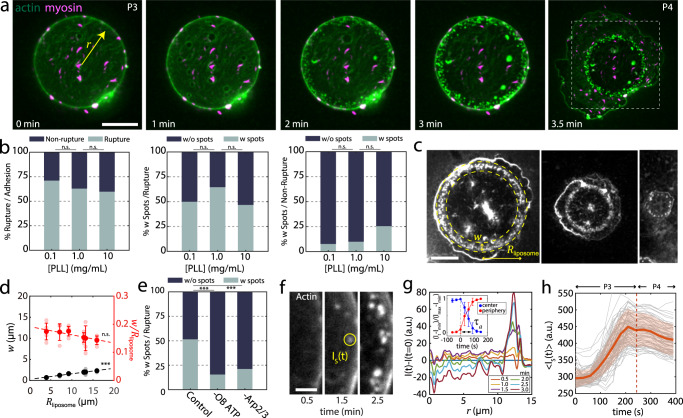
Tension-induced membrane permeability localizes peripheral F-actin assembly. **a** Actin (green) and myosin (magenta) within the liposome adhered to a 2 mg/mL PLL-coated surface. Scale bar, 10 µm. **b** (Left) Percentage of ruptured (gray) and non-ruptured (black) actin liposomes (0.1 mg/ml PLL, *N* = 45; 1 mg/ml PLL, *N* = 27; 10 mg/ml PLL, *N* = 107), (*Middle*) ruptured actin liposomes with spots (gray) and without spots (black) (0.1 mg/ml PLL, *N* = 32; 1 mg/ml PLL, *N* = 17; 10 mg/ml PLL, *N* = 64), (Right) non-ruptured actin liposomes with spots (gray) and without spots (black) (0.1 mg/ml PLL, *N* = 13; 1 mg/ml PLL, *N* = 10; 10 mg/ml PLL, *N* = 43). Independent experiments: *n* = 1 at 0.1 mg/ml PLL, *n* = 2 at 1 mg/ml PLL, *n* = 3 at 10 mg/ml PLL. **c** Fluorescent actin images of burst liposomes with spots. Yellow arrows indicate the spot-ring width *w* and the liposome radius *R*_liposome_. Scale bar, 10 µm. **d** Liposome size-dependence of the *w* and *w*/*R*_liposome_. **e** Percentage of ruptured actin liposomes with spots (gray) without spots (black) in control, without ATP in the outer buffer, and without Arp2/3, at 10 mg/ml PLL (Control, *N* = 31; -OB ATP, *N* = 96; -Arp2/3, *N* = 75; *n* = 2 for each condition). **f** Spot identification of peaks in actin fluorescence intensity. Scale bar, 2 µm. **g** Radial actin fluorescence intensity over time, $r$ is the distance from the liposome center. The intensity was measured 360 times by rotating the radius and averaged. (Inset) Change in intensity at the liposome center (blue) and peripheral spots (red) over time, ${\tau }_{a}$ is the characteristic depolymerization time (*N* = 6, *n* = 3 independent experiments). Time at 0 s corresponds to the onset of depolymerization. **h** Actin fluorescence intensity for the spots over time (N_tot_ = 72). The large standard deviation is due to the variation in the spot growth onset. Error bars are mean ± standard deviation. *** stands for *p* < 0.001. n.s., not significant.

Second, we observe small regions of enhanced fluorescence of actin, near the inner periphery of the adherent liposome, what we term “spots” (Fig. 4a, Supplementary Movies 5 and 6). They reflect polymerization in patches, reminiscent of Arp2/3 growth on liposome surfaces^21,38^. As with clusters, not all liposomes exhibit spots (Fig. 4b, left and middle, 47% ruptured AL, *N* = 64 at 10 mg/mL PLL). Spots are smaller in size than clusters, at approximately 1 μm in diameter. They are densely packed, located within approximately 2 μm from the contact line (Fig. 4c, d). The growth in intensity is coincident with that of the clusters, also remaining after rupture (Supplementary Fig. 7). In the absence of Arp2/3, cofilin or gelsolin, when blisters are present, the prevalence of clusters and spots significantly decreased (Figs. 3h and 4e, Supplementary Fig. 8, and Supplementary Movie 7).

The enhancement of actin fluorescence intensity of both clusters and spots occurs simultaneously with a decrease in actin fluorescence intensity across the inner surface of the liposome (Fig. 4f–h). Overall, this translates to a decrease in actin at the center of the liposome and an accumulation at the periphery, occurring over a timescale ${\tau }_{a}$, of 100 $\pm$ 14 s (Fig. 4g, inset). This suggests the net effect of disassembly of F-actin central to the liposome, and re-assembly near the contact line, and a net radially outwards flux of actin. This timescale is consistent with the timescale of Arp2/3 polymerization outside of liposome in assembly assays^46,47^.

As the growth of the spots occurs micrometers within the periphery of the liposome (Fig. 4c, d), where stresses are concentrated by TFM (Fig. 1e), one hypothesis to explain their assembly is that the polymerization of F-actin by Arp2/3 is related to the accumulation of mechanical stress. However, the impact of mechanical stress on polymerization may be direct, in terms of force-dependent polymerization of F-actin, or indirect through mechanically mediated chemical and diffusive effects. More specifically, the elevated membrane tension may propagate stress or strain to the Arp2/3 complex and impact the assembly of the F-actin. This may suggest potential mechano-sensitivity of the nucleating proteins (Hypothesis 1). Second, prior to the lysis, the liposome becomes transparent, indicative that the concentrations of sucrose and glucose have equilibrated, and there is no longer a difference in the index of refraction across the membrane (Supplementary Figs. 9 and 10). This is evidence of the mechanically-induced permeability of the membrane prior to lysis^48^, which allows ATP to enter the liposome by diffusion at the contact line. The actin may therefore repolymerize within a distance inward from the contact line set by a balance of reaction and diffusion (Hypothesis 2).

To test Hypothesis 1, we estimate length-scales associated with stress propagation to the length over which actin spots form away from the contact line (*L* ~ 2 $\mu m$). To test Hypothesis 1, we assume that the liposome is in mechanical equilibrium, where the surface tension ($\gamma$) is balanced by the lateral interfacial tensions^49^. In this case, the surface tension pulls upwards on the liposome at the contact line, attempting to ‘peel’ the liposome from the surface. At the contact line, the membrane is therefore bent away from the surface to an extent determined by the membrane bending stiffness ${k}_{b}$. The length over which bending is significant is given by the “bendo-capillary” length^50^:

1 $${L}_{{{{{{\rm{b}}}}}}} \sim {\left(\frac{{k}_{{{{{{\rm{b}}}}}}}}{\gamma }\right)}^{1/2}$$

If the bending stiffness reflects that of an actin layer, ${k}_{{{{{{\rm{b}}}}}}}\sim 4{\times 10}^{-18}{J}$^51–54^ (which is two orders of magnitude larger than that of a bilayer membrane^22,24^), and a lysis tension of 0.3–5.0 mN/m, ${30\, < \,L}_{{{{{{\rm{b}}}}}}}\, <$ 100 ${{{{{\rm{nm}}}}}}$. As ${L}_{{{{{{\rm{b}}}}}}}\ll L$, the surface tension of the membrane cannot propagate the distance over which enhanced actin polymerization is observed.

To test Hypothesis 2, we evaluate the prevalence of spots in liposomes that have rupture versus those that not. As the ATP may have been depleted upon the time in which the liposome is first observed, access to ATP from the outside buffer (OB) may be the source of actin repolymerization. Indeed, we find that spots are significantly more prevalent if the liposome has ruptured, suggesting membrane permeability and access to ATP from ADP actin (Fig. 4b, middle and right). Further, we evaluate the prevalence of liposomes that have ruptured, although comparing to conditions in which ATP is present in the OB than to cases in which no ATP is available in the OB (Fig. 4e). Again, we see that spots are consistent with the presence of ATP outside of the liposome. We therefore suggest that membrane tension and tension-induced membrane permeability couples to ATP-reaction-diffusion to induce the formation of spots.

We next test whether the length scale observed $L$, is consistent with a reaction diffusion mechanism by calculation of the Thiele length, ${L}_{{{{{{\rm{Th}}}}}}}$, a characteristic length scale over which reaction occurs (Supplementary Note 2, Supplementary Figs. 11 and 12). By using published values of first order actin-ATP reaction rates, and estimated diffusivities of ATP within actin networks, we find, $1 \, < \, {L}_{{{{{{\rm{Th}}}}}}} \, < \, 10\,\mu m$. Thus, ${L}_{{{{{{\rm{Th}}}}}}}\sim L$, suggesting that the liposomes are in a diffusion-limited condition and spots could be formed by diffusion-limited ATP-driven polymerization.

### Active contractility and cortical viscosity set the rate of pore expansion

**Fig. 5:**
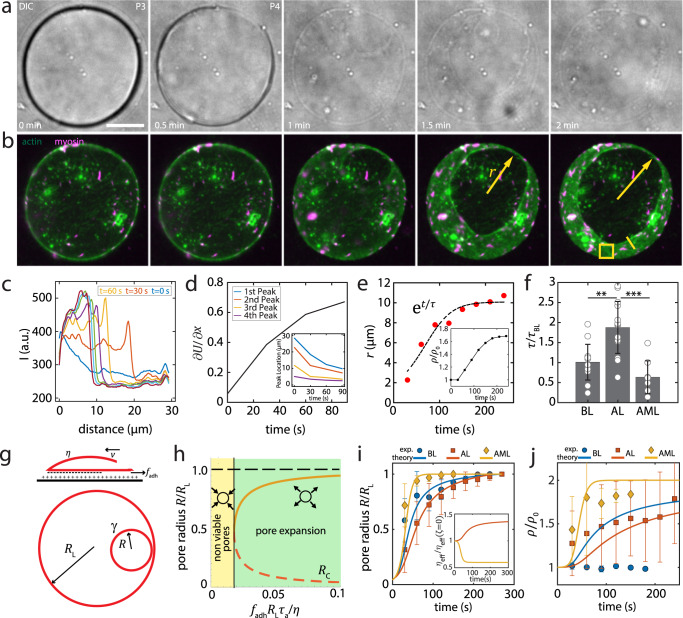
Pore expansion induces myosin-dependent actin flows. **a** Transmitted light and, **b** Fluorescent actin (green) and myosin (magenta) during adhesion and rupture on 2 mg/mL PLL-coated coverslips. Yellow arrow indicates pore radius, *r*. Scale bar, 10 µm. **c** Linescan of fluorescence intensity (I) in (**b**) (solid yellow line) over time. **d** Displacement gradient ($\partial U/\partial x$) of actin from (**c**) and the location of pore boundary (inset). **e** Pore radius over time and exponential fit of a characteristic timescale, τ. Inset: Density ($\rho /{\rho }_{0}$) of actin in (**b**) (yellow square) over time. **f** τ, for bare liposomes (*N*_tot_ = 12; independent experiments, *n* = 9), actin liposomes (*N*_tot_ = 14, *n* = 12), and actomyosin liposomes (*N*_tot_ = 8, *n* = 8). **g** Schematic of pore expansion. Top: side view. Bottom: top view. The red color represents composite actin/membrane layer. **h** Spontaneously formed small pore expands when the adhesive force is higher than a critical value (solid black line). Dashed lines: unstable fixed points for pore radius (*R*). Solid orange line: the steady state pore radius (stable fixed point). **i** Dynamics of pore radius obtained from active gel theory (lines), fitted to experimental data (points). (Inset) The effective viscosity (${\eta }_{{{{{{\rm{eff}}}}}}}$) divided by the effective viscosity with $\zeta =0$ over time. **j** Normalized density of actin layer predicted by theory. Error bars are mean ± standard deviation. ** and *** stand for *p* < 0.01 and 0.001, respectively.

In P4, the liposome ruptures, after initiation of a pore at the contact line (Fig. 5a, Supplementary Movie 8). The area of the pore grows, as it expands across the upper surface of the bilayer, until it is equal to the contact area of the liposome. Upon initial pore formation, the brightfield contrast is lost, reflecting an equilibration of solute that diminishes the difference in refractive indices between inside and outside, and indicating loss of the integrity of the membrane. The bilayer retracts quickly, radially outwards, towards the contact line (Fig. 5b). For actin and actomyosin liposomes, the actin retracts with an altered spatial distribution of intensity (Fig. 5c). Initially homogeneous, the actin intensity at the leading edge of the pore elevates in time, in contrast to actin more distal to the pore. As a result, there is an increasing gradient in actin density and displacement (Fig. 5d). The overall density of actin also increases, as can be seen in the 1.6-fold increase in fluorescence intensity distal to the pore (Fig. 5e, inset).

Cortical F-actin, a sub-membranous actin layer has been shown to slow the rate of spreading during P1^24^. Here, we explore the impact of the actomyosin layer on the mechanics of pores which open due to a relaxation of strong adhesive stresses. Though pore dynamics has been extensively studied for bare liposomes^55,56^, the effect of actin cortical tension is not clear. Fitting the radius of the pore as a function of time^57^ yields a characteristic time scale, τ, of pore opening (Fig. 5e). We observe that the average τ ~ 20 s, whereas that of liposomes with an actin layer is ~80 s (Fig. 5f). In the presence of myosin within the actin layer, the rupture rates decrease to ~20 s. In this case, the membrane has lost integrity, thereby ATP has re-entered the liposome and myosin molecular motors are active as evidenced by enhanced dynamics of myosin motion (Supplementary Fig. 10, Supplementary Movie 9) As there are variations in time scales between experiments, we normalize all $\tau$ to the $\tau$ for the empty liposome, ${\tau }_{{{{{{\rm{BL}}}}}}}$.

From these initial observations, we hypothesize that myosin may increase the rate of pore opening (expansion of the hole), through changing the mechanical properties of the actin layer within the liposome. Previous models have explained pore expansion through a balance of line and surface tension^56^, the rate of which is governed by the ratio of tension to a constant viscosity. However, in the present study, pore opening increases actin density (*ρ*), which may have contrasting effects to increase viscosity and to concentrate active stress. Separately, there is a competition between the turnover of actin (${\tau }_{a}$) and the timescales involved in viscous relaxation, stress generation and friction. Thus, the interplay between these variables resist description by a passive model, and the effect of active stress on material properties of the actin layer and pore dynamics are unknown.

To understand how actin turnover and myosin activity impact actin layer fluidity and pore opening we developed a continuum active gel model for the actin layer, in terms of the actin velocity field $\vec{v}$, and the density field $\rho$ (Supplementary Note 3). Mass conservation implies the following continuity equation for the actin gel

2 $$\frac{\partial \rho }{\partial t}+\nabla .(\rho \vec{v})=-\frac{1}{{\tau }_{{{{{{\rm{a}}}}}}}}(\rho -{\rho }_{0})$$

where ${\tau }_{{{{{{\rm{a}}}}}}}^{-1}$ is the actin turnover rate and ${\rho }_{0}$ is the equilibrium actin density. The mechanics of the membrane-actin layer is characterized by a viscosity $\eta$, an internal pressure $P$ and an active contractile stress ${\sigma }_{{{{{{\rm{a}}}}}}}$, and a membrane tension $\gamma$ at the pore boundary (Fig. 5g). At mechanical equilibrium, local gradients in internal stress of the cortex are balanced by the forces of adhesion (${f}_{{{{{{\rm{adh}}}}}}}$) and friction with the surface underneath, given by

3 $$\nabla .[\eta \nabla \vec{v}+{\sigma }_{{{{{{\rm{a}}}}}}}(\rho ){{{{{\boldsymbol{I}}}}}}-P(\rho ){{{{{\boldsymbol{I}}}}}}]=\Gamma \vec{v}-{\vec{f}}_{{{{{{\rm{adh}}}}}}}$$

where $\Gamma$ is a friction coefficient. Using the above equations, we can determine the dynamics of a circular pore of radius $R(t)$ in the active gel (see Supplementary Note 3, Supplementary Table 1). During the initial phase of pore opening, dynamics of the pore radius can be approximated as:

4 $$\frac{{dR}}{{dt}}\approx [(\sigma _{0}+2{f}_{{{{{{\rm{adh}}}}}}}{R}_{{{{{{\rm{L}}}}}}}/3)R-2\gamma ]/{\eta }_{{{{{{\rm{eff}}}}}}}$$

where ${\eta }_{{{{{{\rm{eff}}}}}}}$ is the effective viscosity of the active gel, given by ${\eta }_{{{{{{\rm{eff}}}}}}}=\eta +(\eta -2{\rho }_{0}{\tau }_{{{{{{\rm{a}}}}}}}\zeta ){R}^{2}/{R}_{{{{{{\rm{L}}}}}}}^{2}$, ${\sigma }_{0}={\sigma }_{{{{{{\rm{a}}}}}}}(\rho ={\rho }_{0})$ is the contractile stress, and ${R}_{{{{{{\rm{L}}}}}}}$ is the radius of the liposome. Here $\zeta$ is defined as $\zeta =\frac{d}{d\rho }({\sigma }_{{{{{{\rm{a}}}}}}}-P){|}_{\rho ={\rho }_{0}}$. The rate of pore opening is inversely proportional to the effective viscosity ${\eta }_{{{{{{\rm{eff}}}}}}}$. In an actin liposome $\zeta\, < \,0$ and ${\eta }_{{{{{{\rm{eff}}}}}}}\, > \,\eta$. Conversely, in an actomyosin gel $\zeta\, > \,0$ and ${\eta }_{{{{{{\rm{eff}}}}}}}\, < \,\eta$, implying that myosin fluidizes the actin layer promoting faster pore expansion.

Our continuum theory predicts that pore expansion occurs above a critical adhesive force for pores with initial size larger than a critical radius ${R}_{{{{{{\rm{c}}}}}}}=6\gamma /(3\sigma _{0}+2{f}_{{{{{{\rm{adh}}}}}}}{R}_{{{{{{\rm{L}}}}}}})$ (Fig. 5h). At sufficiently large adhesive forces, the critical pore size for rupture, ${R}_{{{{{{\rm{c}}}}}}}$, is very small. Over time, these small pores would eventually reach the liposome size ${R}_{{{{{{\rm{L}}}}}}}$. This is the experimentally relevant regime where spontaneously formed pores in the membrane-cortex layer can start expanding to eventually rupture the liposome.

The force balance and inclusion of density dependent active stresses (Eqs. (3) and (4)) reproduces the pore expansion dynamics as measured in experiments (Fig. 5i). The timescale associated with pore expansion depends on the effective viscosity (${\eta }_{{{{{{\rm{eff}}}}}}}$) of the gel as well as active stress. An enhanced viscosity in actin liposomes (${\eta }_{{{{{{\rm{eff}}}}}}}\, > \,\eta$) results in a slower rate of pore opening compared to bare liposomes, whereas a contractile active stress in actomyosin liposomes results in faster pore expansion due to a lower effective viscosity (${\eta }_{{{{{{\rm{eff}}}}}}}\, < \,\eta$) (Fig. 5i-inset). This finding supports the experimental hypothesis of enhanced actin viscosity slowing down the pore opening. We find that the average actin density in the membrane-cortex layer increases with expanding pore size (Fig. 5j), consistent with experimental data (Fig. 5e-inset). This results from a decrease in liposome surface area with pore expansion. We note that the density of lipids for BL is constant over time in the experiment but is increased in theory, possibly due to the leakage of lipids from the liposome to the bottom substrate during the pore opening (Supplementary Fig. 13), which is not considered in the present model. Theoretical fitting to the pore opening of actomyosin liposome gives contribution of myosin to the tension ${\sigma }_{a}d\sim {10}^{-7}$ N/m (*d* is the thickness of the actin layer (Supplementary Fig. 2)), comparable to the estimated tension produced by a single myosin thick filament (Methods). Furthermore, assuming no-slip boundary condition at the pore edge and slip boundary condition at contact line of liposome allows us to predict the spatial patterns of actomyosin flow during pore opening and expansion. The actin flow profile obtained from our theory quantitatively matches the experimentally measured flow profile around the pore (Supplementary Fig. 14). In particular, the flow velocity is non-monotonic in time, and localizes near the pore over a length scale determined by the ratio of actin viscosity to the substrate friction.

## Discussion

Here, we study the impacts of elevated membrane tension on the organization of the actomyosin cytoskeleton enclosed in liposomes as a minimal adherent cell model. Upon adhesion and spreading of liposomes, we observe a localized change in F-actin intensity. At later times upon membrane lysis, the timescale of pore expansion slows down in the presence of the actin layer, whereas the inclusion of myosin accelerates the pore expansion. These results indicate that passive and active stresses coordinate to dramatically impact the cell-scale architecture and dynamics of the cytoskeleton.

In considering the accumulation of membrane tension during adhesion, the assembly of proteins that constitute the focal adhesion-cytoskeletal complex, which is responsible for mediating cell-ECM adhesion and transmitting mechanical stress to the cytoskeleton is abstracted by charge-based adhesion between the membrane and the surface, and direct coupling of the membrane to Arp2/3 nucleation factors. In making this abstraction, the number of components is reduced and the nature of their interaction is simplified. This enables isolation and highlighting of the roles of mechanics (e.g. surface tensions) on the assembly and dynamics of encapsulated F-actin within the liposome. Thus, this study explores the effect of accumulated stress on the actin cytoskeleton, without regards to the nature of coupling of the membrane to the ECM nor the membrane to the actin.

At early stages in P3, we observe an altered spatial pattern of actin assembly and disassembly. In the center of the liposome, fluorescence decreases. By contrast, at the periphery, fluorescence increases. In this case, the actin network is also transformed; formerly isotropic actin is preferentially re-assembled in the form of clusters and spots. However, the origin of altered polymerization is unclear and may be due to multiple effects. Here, we present and discuss hypotheses for their assembly.

First, spots are localized near the contact line, like the localization of Arp2/3 polymerization at the periphery of the cell^58,59^. To explain the mechanism of peripherally localized spot formation, we proposed hypothesis (1) mechano-sensitivity of the nucleating proteins and hypothesis (2) mechanically-induced permeability of the membrane prior to lysis. Hypothesis (1) is based on the observation that TFM showed stresses are concentrated within the periphery of the liposome. To date, stresses as low as approximately 25 Pa have been shown to increase Arp2/3 density and mechanical stiffness in vitro^60^. However, the direct impact of mechanical stress on Arp2/3 is unlikely due to the bendo-capillary length being much smaller than the distance into the liposome at which polymerization is altered. On the other hand, hypothesis (2) is based on the observation that liposomes become transparent prior to lysis, suggesting the mechanically-induced permeability of the membrane that allows ATP to enter the liposomes at the contact line. Therefore, actin may repolymerize within a distance inward from the contact line set by a balance of reaction and diffusion. We have tested this hypothesis by observing the prevalence of actin polymerization events when external ATP is present. We have also observed that spots are significantly more prevalent when the liposome has ruptured (indicating access to external ATP). Finally, we also estimate the characteristic reaction-diffusion length scale called Thiele length. The series of experimental and theoretical tests support the hypothesis (2). A previous study on the spreading of bare liposome on PLL-coated substrates proposed a permeation mechanism at the contact line during spreading^48^. Thus, in this case, mechanical forces may couple to a reaction-diffusion mechanism that impacts F-actin assembly. However, to further validate hypothesis (2), further characterizations, altering diffusion rates and including an ATP regeneration system, could be performed in future studies.

Second, we note that clusters are spatially associated with inward basal protrusions (‘blisters’). We speculate that the formation of blisters may exclude lipids containing VCA domain of WASP, thereby aggregating Arp2/3 at the perimeter of the blisters. This exclusion may further create a convex surface that preferentially assembles Arp2/3 branches as shown in vitro^61^ or instead involve the direct sensing of membrane curvature as seen in vivo^62^. Of note, it has been shown that dimerization of VCA increases its affinity for Arp2/3 complex by more than 100-fold and greatly enhances the actin assembly^63^. Thus, in this case, membrane deformation may couple to F-actin assembly. Indeed, a study has reported membrane curvature sensing of the F-actin network polymerized by the Arp2/3 complex in liposomes^64^. However, we also note that some of the clusters can occur after the disappearance of blisters. Thus, the relationship between curvature and assembly is unclear. Instead, tension-induced permeability might play a role in the assembly of clusters as it does in spots. Membrane tension at the blister may be elevated when it expands; as a result, the membrane at the blister might become permeable, allowing external ATP to enter the liposome at the blister. Thus, in this case, actin may repolymerize around the blister by a balance of reaction and diffusion of ATP. To date, the exact mechanism of the cluster formation is not fully clear; thus, further investigations, such as controlling the size and the number of blisters and theoretical modeling^41^, will be essential future works.

Finally, we consider the accumulation of mechanical stresses during rupture and pore expansion in P4. During this process, the membrane tension is decreased in comparison to tension within the actin layer. The pore expands, leading to inhomogeneous actin density and actin velocity. However, in the presence of myosin, there is additional tension within the actin layer (Supplementary Figs. 4 and 5), akin to cortical tension that exists within eukaryotic cells. In this case, the expansion of the pore occurs faster in the presence of myosin than in its absence. We suggest this is due to myosin-generated active stress that induces contractility on the order of seconds (Supplementary Fig. 14)^32,65,66^. Based on this experimental hypothesis, our modeling indicates that this has the effect of an effective decrease in viscosity, driven by active stress. Furthermore, it shows that the dynamics of pore expansion are explicitly a balance of this viscosity with the friction between the liposome and the substrate. It should be noted that pore opening in lipid vesicles has been used as a theoretical model to study cell rupture upon bacterial infection into endothelial cells^67,68^, in which ‘tunnels’ are created in the cytoskeleton.

Passive stresses drive F-actin reorganization through induced membrane permeability that couples to reaction and diffusion processes. By contrast, when passive stresses are relaxed, active stresses drive the dynamics of pore opening. Thus, these two forces coordinate complex cell-like organizations and dynamics of the F-actin cytoskeleton.

## Methods

### Experimental design: lipid composition

Liposomes are formed with a combination of neutral L-α-phosphatidylcholine from egg yolk (EPC, 840051 C, 53%), cholesterol (ovine wool, 110796, 36%), and 1,2-dioleoyl-sn-glycero-3- [n(5-amino-1-carboxypentyl) iminodiacetic acid]succinyl nickel salt (DOGS-NTA-Ni, 10%). Lipids were purchased from Avanti Polar Lipids (Alabaster, AL). For fluorescent contrast, we use Oregon Green or Texas Red 1,2-Dihexadecanoyl-snGlycero-3-Phosphoethanolamine (DHPE) from Molecular Probes (Invitrogen) at 1%. OG 488 nm DHPE has a pKa of 4.7, and thus has negative charge at experimental pH. For detailed lipid preparation, see Supplementary Note 1.

### Substrate preparation

For adhesion on coverslips, 50 µl of 0.1–10 mg/mL Poly-L-lysine Hydrobromide (>70k MW, MP Biomedicals, or 70k-150k MW, Sigma-Aldrich) is mixed with 7.5% sodium bicarbonate to achieve a pH 9, and then is placed on coverslip within a chamber, and sandwiched with another coverslip which was treated with Rain-X to spread the PLL across the entire surface for 15 min. The coverslip is then rinsed with water, and then with O-buffer.

For high adhesion on polyacrylamide gels, the gels are polymerized onto cleaned coverslip surfaces. The coverslips are treated with glutaraldehyde and then aminopropyl silane which will react with the acrylamide. Different concentrations of bis/acrylamide are mixed with 0.1 mg/mL ammonium persulfate to yield a gel with variable Elastic Modulus (ν = 0.5). Within the gel, 40 nm far red (647 nm) beads are embedded prior to polymerization. 12 µl of gel solution is then added to the coverslip and covered with a Rain-X-treated coverslip to make it hydrophilic. After the gels are polymerized, they are reacted with by the Sulfo-Sanpah protocol as previously published^25^. The surface of the reactive gels is then coated with poly-L-Lysine Hydrobromide of »70k MW (MP Biomedicals) which has been re-suspended in MilliQ water, and the pH set to 9. The reaction proceeds for 1 hour in the dark, and the coverslips are then rinsed in 1X PBS.

### Protein concentrations

The final concentration of actin and myosin within the liposomes is 5.7 µM and 50 nM respectively. VCA was used at 0.64 μM. Arp2/3 for actin polymerization was at 0.12 µM. Gelsolin and Cofilin severing proteins were at 50 nM and 2 µM respectively. Details of buffer conditions are available in the Supplementary Note 1.

### Tension from myosin thick filaments

Within a thick filament approximately 1 μm in size, based on a duty ratio of 4-10%^69,70^ there are a minimum of 12 dimers bound at any given time^65^. As a single motor can generate approximately 3.4 pN of force under isometric conditions^71^, and thus each thick filament can generate a minimum force of 44 pN. Thus, for a 10 μm radius liposome, the contribution to the tension from a thick filament is approximately ~10^-7 ^N/m, comparable to typical floppy membrane tension^72^. This explains the changes in liposome shape for low thick filament density when non-adherent (Supplementary Figs. 4 and 5) and the approximate contribution to tension during pore opening (Fig. 4).

### Microscopy

Images were acquired by either Nikon Ti inverted microscope equipped with a 60× 1.4-NA oil immersion lens (Nikon), a spinning-disk confocal (CSUX; Yokagawa), and CCD camera (Coolsnap HQ2; Photometrics) controlled by Metamorph (MDS Analytical Technologies), or Leica DMi8 inverted microscope equipped with a 63× 1.4-NA oil immersion lens (Leica Microsystems), a spinning-disk confocal (CSU22; Yokagawa), and sCMOS camera (Zyla; Andor Technology) controlled by Andor iQ3 (Andor Technology).

### Statistics and reproducibility

Number of experiments performed are specified in Supplementary Table 2. Statistical tests comparing distributions are done with a two-sided *t* test. Statistical tests comparing bar plots are done with Fisher’s exact test. All data displayed as a single value with an error bar is quoting the mean ± standard deviation. The symbols *, **, and *** represent *p* < 0.05, 0.01, and 0.001 respectively. Fitted lines are shown to reject the null hypothesis to an extent that depends on the quoted *p* value.

### Reporting summary

Further information on research design is available in the Nature Portfolio Reporting Summary linked to this article.

## Supplementary information

Supplementary Information

Description of Additional Supplementary Files

Supplementary Movie 1

Supplementary Movie 2

Supplementary Movie 3

Supplementary Movie 4

Supplementary Movie 5

Supplementary Movie 6

Supplementary Movie 7

Supplementary Movie 8

Supplementary Movie 9

Supplementary Data 1

Reporting Summary

## Data Availability

The data supporting this manuscript are available in Supplementary Data 1 and from the corresponding author on reasonable request.

## References

[1] Salbreux G, Charras G, Paluch E (2012). Actin cortex mechanics and cellular morphogenesis. Trends Cell Biol..

[2] Murrell M, Oakes PW, Lenz M, Gardel ML (2015). Forcing cells into shape: the mechanics of actomyosin contractility. Nat. Rev. Mol. Cell Biol..

[3] Bray D, White JG (1988). Cortical flow in animal cells. Science.

[4] Munro E, Nance J, Priess JR (2004). Cortical flows powered by asymmetrical contraction transport PAR proteins to establish and maintain anterior-posterior polarity in the early C. elegans embryo. Dev. Cell.

[5] Chugh P (2017). Actin cortex architecture regulates cell surface tension. Nat. Cell Biol..

[6] Fritzsche M, Erlenkamper C, Moeendarbary E, Charras G, Kruse K (2016). Actin kinetics shapes cortical network structure and mechanics. Sci. Adv..

[7] Gauthier NC, Masters TA, Sheetz MP (2012). Mechanical feedback between membrane tension and dynamics. Trends Cell Biol..

[8] Charras GT, Yarrow JC, Horton MA, Mahadevan L, Mitchison TJ (2005). Non-equilibration of hydrostatic pressure in blebbing cells. Nature.

[9] Lieber, A. D., Yehudai-Resheff, S., Barnhart, E. L., Theriot, J. A. & Keren, K. Membrane tension in rapidly moving cells is determined by cytoskeletal forces. *Curr. Biol.* **23**, 1409–1417 (2013). 10.1016/j.cub.2013.05.063 23831292

[10] Raucher D, Sheetz MP (2000). Cell spreading and lamellipodial extension rate is regulated by membrane tension. J. Cell Biol..

[11] Houk AR (2012). Membrane tension maintains cell polarity by confining signals to the leading edge during neutrophil migration. Cell.

[12] Liu Y (2012). Constitutively active ezrin increases membrane tension, slows migration, and impedes endothelial transmigration of lymphocytes in vivo in mice. Blood.

[13] Cuvelier D, Nassoy P (2004). Hidden dynamics of vesicle adhesion induced by specific stickers. Phys. Rev. Lett..

[14] Nardi J, Bruinsma R, Sackmann E (1998). Adhesion-induced reorganization of charged fluid membranes. Phys. Rev. E.

[15] Pontani LL (2009). Reconstitution of an actin cortex inside a liposome. Biophys. J..

[16] Loiseau E (2016). Shape remodeling and blebbing of active cytoskeletal vesicles. Sci. Adv..

[17] Carvalho K (2013). Cell-sized liposomes reveal how actomyosin cortical tension drives shape change. Proc. Natl. Acad. Sci. USA.

[18] Miyata H, Hotani H (1992). Morphological changes in liposomes caused by polymerization of encapsulated actin and spontaneous formation of actin bundles. Proc. Natl. Acad. Sci. USA.

[19] Miyata H, Kinosita K (1994). Transformation of actin-encapsulating liposomes induced by cytochalasin D. Biophys. J..

[20] Miyata H, Nishiyama S, Akashi K, Kinosita K (1999). Protrusive growth from giant liposomes driven by actin polymerization. Proc. Natl. Acad. Sci. USA.

[21] Simon C (2019). Actin dynamics drive cell-like membrane deformation. Nat. Phys..

[22] Guevorkian K, Manzi J, Pontani LL, Brochard-Wyart F, Sykes C (2015). Mechanics of biomimetic liposomes encapsulating an actin shell. Biophys. J..

[23] Maan R, Loiseau E, Bausch AR (2018). Adhesion of active cytoskeletal vesicles. Biophys. J..

[24] Murrell M (2011). Spreading dynamics of biomimetic actin cortices. Biophys. J..

[25] Murrell MP (2014). Liposome adhesion generates traction stress. Nat. Phys..

[26] Cuvelier D (2007). The universal dynamics of cell spreading. Curr. Biol..

[27] Evans E, Rawicz W (1990). Entropy-driven tension and bending elasticity in condensed-fluid membranes. Phys. Rev. Lett..

[28] Evans E, Heinrich V, Ludwig F, Rawicz W (2003). Dynamic tension spectroscopy and strength of biomembranes. Biophys. J..

[29] Kliesch TT (2017). Membrane tension increases fusion efficiency of model membranes in the presence of SNAREs. Sci. Rep..

[30] Chabanon M, Ho JCS, Liedberg B, Parikh AN, Rangamani P (2017). Pulsatile lipid vesicles under osmotic stress. Biophys. J..

[31] Pautot S, Frisken BJ, Weitz DA (2003). Production of unilamellar vesicles using an inverted emulsion. Langmuir.

[32] Murrell M, Thoresen T, Gardel M (2014). Reconstitution of contractile actomyosin arrays. Methods Enzymol..

[33] Sakamoto T, Limouze J, Combs CA, Straight AF, Sellers JR (2005). Blebbistatin, a myosin II inhibitor, is photoinactivated by blue light. Biochemistry.

[34] Linsmeier I (2016). Disordered actomyosin networks are sufficient to produce cooperative and telescopic contractility. Nat. Commun..

[35] Schuppler M, Keber FC, Kroger M, Bausch AR (2016). Boundaries steer the contraction of active gels. Nat. Commun..

[36] Sonal (2018). Myosin-II activity generates a dynamic steady state with continuous actin turnover in a minimal actin cortex. J. Cell Sci..

[37] Carvalho K (2013). Actin polymerization or myosin contraction: two ways to build up cortical tension for symmetry breaking. Philos. Trans. R. Soc. Lond. B Biol. Sci..

[38] Muresan CG (2022). F-actin architecture determines constraints on myosin thick filament motion. Nat. Commun..

[39] Tsai FC, Stuhrmann B, Koenderink GH (2011). Encapsulation of active cytoskeletal protein networks in cell-sized liposomes. Langmuir.

[40] Takiguchi K, Yamada A, Negishi M, Tanaka-Takiguchi Y, Yoshikawa K (2008). Entrapping desired amounts of actin filaments and molecular motor proteins in giant liposomes. Langmuir.

[41] Dinet, C., Torres-Sánchez, A., Arroyo, M. & Staykova, M. Patterning of membrane adhesion under hydraulic stress. bioRxiv 2023.01.04.522479 (2023). 10.1038/s41467-023-43246-7 PMC10656516 37978292

[42] Gordon VD, Deserno M, Andrew CMJ, Egelhaaf SU, Poon WCK (2008). Adhesion promotes phase separation in mixed-lipid membranes. EPL (Europhys. Lett.).

[43] Sun YD, Leong NT, Wong T, Drubin DG (2015). A Pan1/End3/Sla1 complex links Arp2/3-mediated actin assembly to sites of clathrin-mediated endocytosis. Mol. Biol. Cell.

[44] Case LB, Waterman CM (2011). Adhesive F-actin waves: a novel integrin-mediated adhesion complex coupled to ventral actin polymerization. PLoS One.

[45] Bretschneider T (2004). Dynamic actin patterns and Arp2/3 assembly at the substrate-attached surface of motile cells. Curr. Biol..

[46] Goley ED (2010). An actin-filament-binding interface on the Arp2/3 complex is critical for nucleation and branch stability. Proc. Natl. Acad. Sci. USA.

[47] Gat S, Simon C, Campillo C, Bernheim-Groswasser A, Sykes C (2020). Finger-like membrane protrusions are favored by heterogeneities in the actin network. Soft Matter.

[48] Bernard AL, Guedeau-Boudeville MA, Jullien L, di Meglio JM (2000). Strong adhesion of giant vesicles on surfaces: Dynamics and permeability. Langmuir.

[49] de Gennes P-G, Brochard-Wyart F., Quéré D. Capillarity: Deformable Interfaces. In: *Capillarity and Wetting Phenomena: Drops, Bubbles, Pearls, Waves* (ed^(eds). Springer New York (2004).

[50] Boddeker TJ (2022). Non-specific adhesive forces between filaments and membraneless organelles. Nat. Phys..

[51] Helfer E (2000). Microrheology of biopolymer-membrane complexes. Phys. Rev. Lett..

[52] Hackl W, Barmann M, Sackmann E (1998). Shape changes of self-assembled actin bilayer composite membranes. Phys. Rev. Lett..

[53] Simson R (1998). Membrane bending modulus and adhesion energy of wild-type and mutant cells of Dictyostelium lacking talin or cortexillins. Biophys. J..

[54] Manoussaki D, Shin WD, Waterman CM, Chadwick RS (2015). Cytosolic pressure provides a propulsive force comparable to actin polymerization during lamellipod protrusion. Sci. Rep..

[55] Brochard-Wyart F, de Gennes PG, Sandre O (2000). Transient pores in stretched vesicles: role of leak-out. Phys. A.

[56] Sandre O, Moreaux L, Brochard-Wyart F (1999). Dynamics of transient pores in stretched vesicles. Proc. Natl Acad. Sci. USA.

[57] Brochard-Wyart F, de Gennes PG (2002). Adhesion induced by mobile binders: dynamics. Proc. Natl Acad. Sci. USA.

[58] Kelleher JF, Atkinson SJ, Pollard TD (1995). Sequences, structural models, and cellular localization of the actin-related proteins Arp2 and Arp3 from Acanthamoeba. J. Cell Biol..

[59] Machesky LM (1997). Mammalian actin-related protein 2/3 complex localizes to regions of lamellipodial protrusion and is composed of evolutionarily conserved proteins. Biochem J..

[60] Bieling P (2016). Force feedback controls motor activity and mechanical properties of self-assembling branched actin networks. Cell.

[61] Risca VI (2012). Actin filament curvature biases branching direction. Proc. Natl. Acad. Sci. USA.

[62] Lou HY (2019). Membrane curvature underlies actin reorganization in response to nanoscale surface topography. Proc. Natl. Acad. Sci. USA.

[63] Padrick SB (2008). Hierarchical regulation of WASP/WAVE proteins. Mol. Cell.

[64] Baldauf, L., Frey, F., Perez, M. A., Idema, T., Koenderink, G. H. Reconstituted branched actin networks sense and generate micron-scale membrane curvature. bioRxiv, 2022.2008.2031.505969 (2022).

[65] Murrell M, Gardel ML (2014). Actomyosin sliding is attenuated in contractile biomimetic cortices. Mol. Biol. Cell.

[66] Murrell MP, Gardel ML (2012). F-actin buckling coordinates contractility and severing in a biomimetic actomyosin cortex. Proc. Natl. Acad. Sci. USA.

[67] Boyer L (2006). Induction of transient macroapertures in endothelial cells through RhoA inhibition by Staphylococcus aureus factors. J. Cell Biol..

[68] Gonzalez-Rodriguez D (2012). Cellular dewetting: opening of macroapertures in endothelial cells. Phys. Rev. Lett..

[69] Harris DE, Warshaw DM (1993). Smooth and skeletal muscle myosin both exhibit low duty cycles at zero load in vitro. J. Biol. Chem..

[70] Johnson CA (2019). The ATPase cycle of human muscle myosin II isoforms: Adaptation of a single mechanochemical cycle for different physiological roles. J. Biol. Chem..

[71] Finer JT, Simmons RM, Spudich JA (1994). Single myosin molecule mechanics: piconewton forces and nanometre steps. Nature.

[72] Betz T, Lenz M, Joanny JF, Sykes C (2009). ATP-dependent mechanics of red blood cells. Proc. Natl. Acad. Sci. USA.

