# Author Correction: Membrane tension induces F-actin reorganization and flow in a biomimetic model cortex

**DOI:** 10.1038/s42003-023-04818-x

**Published:** 2023-04-28

**Authors:** Ryota Sakamoto, Deb Sankar Banerjee, Vikrant Yadav, Sheng Chen, Margaret L. Gardel, Cecile Sykes, Shiladitya Banerjee, Michael P. Murrell

**Affiliations:** 1 Department of Biomedical Engineering, Yale University, 10 Hillhouse Avenue, New Haven, CT USA; 2 Systems Biology Institute, 850 West Campus Drive, West Haven, CT USA; 3 Department of Physics, Carnegie Mellon University, Pittsburgh, PA 15213 USA; 4 Department of Physics, University of Chicago, Chicago, IL 60637 USA; 5 James Franck Institute, University of Chicago, Chicago, IL 60637 USA; 6 Institute for Biophysical Sciences and Pritzker School of Molecular Engineering, University of Chicago, Chicago, IL 60637 USA; 7 Laboratoire de Physique, l’Ecole Normale Supérieure, Paris, France; 8 Department of Physics, Yale University, 217 Prospect Street, New Haven, CT USA

**Keywords:** Permeation and transport, Membrane biophysics, Biophysics

Correction to: *Communications Biology* 10.1038/s42003-023-04684-7, published online 27 March 2023.

In the original version of the Article, Author ‘Margaret L. Gardel’ name was missing middle initial. Additionally, two affiliations for this author were omitted (James Franck Institute, University of Chicago, Chicago, IL 60637, USA and Institute for Biophysical Sciences and Pritzker School of Molecular Engineering, University of Chicago, Chicago, IL 60637, USA).

Corrected list of authors and affiliations:

Ryota Sakamoto^1,2^, Deb Sankar Banerjee^3^, Vikrant Yadav^1,2^, Sheng Chen^1,2^, Margaret L. Gardel^4,5,6^ Cecile Sykes^7^, Shiladitya Banerjee^3^ & Michael P. Murrell^1,2,8^

^1^Department of Biomedical Engineering, Yale University, 10 Hillhouse Avenue, New Haven, CT, USA.

^2^Systems Biology Institute, 850 West Campus Drive, West Haven, CT, USA.

^3^Department of Physics, Carnegie Mellon University, Pittsburgh, PA 15213, USA.

^4^Department of Physics, University of Chicago, Chicago, IL 60637, USA.

^5^James Franck Institute, University of Chicago, Chicago, IL 60637, USA.

^6^Institute for Biophysical Sciences and Pritzker School of Molecular Engineering, University of Chicago, Chicago, IL 60637, USA

^7^Laboratoire de Physique, l’Ecole Normale Supérieure, Paris, France.

^8^Department of Physics, Yale University, 217 Prospect Street, New Haven, CT, USA.

Further, in this Article the grant number NSF DMR 2215605 related to author M.L.G. was omitted from the “Acknowledgements” section.

Corrected Acknowledgements:

We would also like to thank Dr. Robert Style, Professor Eric Dufresne, and Professor Kate Jensen for constructive discussions. The authors acknowledge funding ARO MURI W911NF‐14‐1‐0403 to M.G. and M.P.M, NIH RO1 GM126256 to V.Y. and M.P.M, NIH U54 CA209992 to M.P.M, and Human Frontier Science Program (HFSP) grant # RGY0073/2018 to S.B. & M.P.M. S.B. acknowledges support from NIH R35 GM143042 and NSF MCB-2203601. M.L.G. acknowledges NSF DMR 2215605. R.S. acknowledges support from the Overseas Postdoctoral Fellowships of the Uehara Memorial Foundation. Any opinion, findings, and conclusions or recommendations expressed in this material are those of the authors(s) and do not necessarily reflect the views of the NSF, NIH, ARO, or HFSP.

This has now been corrected in both the PDF and HTML versions of the Article.

