# Supplementary material for: Membrane tension induces F-actin reorganization and flow in a biomimetic model cortex
Source: Commun Biol. 2023 Mar 27;6:325. doi: 10.1038/s42003-023-04684-7 (PMC10043271; doi:10.1038/s42003-023-04684-7)
Supplement: Supplementary file 1 — Supplementary Information [file 42003_2023_4684_MOESM1_ESM.pdf]

Supplementary Information for

Membrane Tension Induces F-actin Reorganization and Flow in a Biomimetic Model Cortex

Ryota Sakamoto, Deb Sankar Banerjee, Vikrant Yadav, Sheng Chen, Margaret Gardel, Cecile Sykes, Shiladitya Banerjee, and Michael P. Murrell*

*Corresponding author: **Email**: michael.murrell@yale.edu

This PDF file includes:

Supplementary Notes 1-3
Figures S1 to S14
Tables S1, S2
Supplementary References 1-14

Supplementary Note 1: Experimental Design

Lipid Preparation

After preparing the lipids as described in the materials and methods sections, the lipids are combined in a glass vial and dried under N_2 gas. The chloroform is dried, and the lipids are dissolved in mineral oil (Sigma). The oil mixture is then sonicated in a bath sonicator for 10-30 minutes at room temperature. The oil is heated to 50°C for 3 hours. The mixture is then cooled to room temperature and stored at 4°C. Then 5 µL of internal buffer is added to 250 µL of mineral oil in a 0.5 mL eppendorf. This mixture is then syringed in a glass syringe (Hamilton) 1-2 times. Separately, in a low absorption 0.5 mL eppendorf, 30 µL of mineral oil is added to the top of 70 µL of external buffer (see below). Then, the emulsion is added to the top of the mineral oil layer in the low-absorption eppendorf. This mixture is then centrifuged at 100g for 15 minutes at 4°C.

Buffer Preparation

Separate buffers are created for the storage and purification of myosin, as well as for the buffers to store and polymerize F-actin and enable its associated proteins. The following buffers are added to a final buffer (FB) which is used to encapsulate within the inverted emulsion.

- Myosin Storage Buffer (MSB): is 0.5 M KCl and 0.1 M HEPES. The stock concentration of the skeletal muscle myosin (Cytoskeleton) is 10 µM. This is the buffer myosin is stored in as dimer, after it has been fluorescently labelled. Myosin assembly is described in detail in previous publications (28).
- Internal Polymerization (IP) Buffer: 80 mM KCl, 3.2 mM $CaCl_2$, 3.2 mM $MgCl_2$, 8 mM HEPES, 0.8 mM DTT, 0.4 mM DABCO, 8 mM ATP, 80 mg/ml dextran, 280 mM sucrose.
- Protein mix (PM): Protein is added at the following concentrations: 5.7 µM actin (Cytoskeleton), 1.3 µM Fluorescent Alexa 568 Actin (Molecular Probes), 0.12 µM Arp2/3, 50 nM Gelsolin (Cytoskeleton), 2 µM Cofilin (Cytoskeleton), 0.64 µM VCA-his (1:10 VCA). In addition, 1 µL of 0.8 M blebbistatin was added to keep the myosin inactive until imaged.
- Internal, Non-Polymerizing (INP Buffer) 0.1 mM $CaCl_2$, 10 mM HEPES, 6 mM DTT, 0.13 µM DABCO, 140 mM Sucrose, 50 mg/ml dextran, 0.2 mM ATP.
- The spin-down buffer (SDB): contains 2 µL dark phalloidin, 4.2 µL 238 µM actin. 40 µL I-buffer. 20 µL myosin (in MSB), 4.3 µL of 4.6 M KCl. Spun at 39,000 rpm for 1 hr at 4C.
- Outside Buffer (OB): 10 mM HEPES (pH 7.5), 2mM $MgCl_2$, 0.2 mM $CaCl_2$, 2mM ATP, 6 mM DTT, 0.13 mM DABCO, 275 mM Glucose, 0.01 mg/ml Casein. The casein is added to minimize liposomes sticking together but does not significantly change adhesion to surface.

Final Buffer (FB): 4 µL INP, 3.25 µL PM, 13.3 µL IP, 2 µL SDB buffer supernatant. This is the buffer that will encapsulate within the liposome. The FB is approximately 250 mOsm. The osmolarity of the OB is adjusted with glucose such that the osmotic pressure difference between OB and FB is between 20 and 60 mOsm.

Thick filament density measurements

The spin-down procedure (Buffer preparation) reduces the concentration of myosin from 240 nM, which we estimate to range between 10-50 nM within the liposome. However, the number of assembled thick filaments formed within the liposome at this concentration varies significantly, between 0 and 40 per liposome. We therefore characterize myosin concentration through visualization of the number density ρ, of thick filaments within a liposome after formation.

Mean-Squared Displacement

First, myosin thick filaments are identified by their peaks in fluorescence using Imaris (Bitplane) spot tracking algorithms. Then, the dynamics of myosin thick filament motion is quantified by the mean-squared displacement (MSD) within a 2D plane. A difference in position within this plane is taken over an elapsed (real) time t, between the initial position of a thick filament, $\vec{r}(0)$, and the displacement over the elapsed time, $\vec{r}(\mathrm{t})$.

$$MSD(t) = \langle \frac{1}{N} \sum_{i=1}^{N} (\vec{r}(0) - \vec{r}_i(\mathrm{t}))^2 \rangle$$

Supplementary Note 2: Thiele Modulus and Thiele Length

The ATP enters the liposome at the contact line, making the plane of the actin network at the bottom gets repolymerized. We assume that the reaction rate of the ATP is rapid and the diffusion force can only carry the ATP (from outside of the liposome) a small distance away from the membrane, which triggers the formation of actin spots near the inner periphery of the adherent liposome.

To verify it, consider the Thiele modulus [1] of the first-order reaction of ATP hydrolysis by polymerized actin [2,3]:

$$\phi_{Th} = r\sqrt{\frac{k_1}{D_{ATP}}}$$

where, k_1 is the reaction rate of first-order reaction ($k_1 = 0.02 \sim 0.3 s^{-1}$ as reported by [2,4-6]), r is the length scale of the source of diffusion (using $r = 10\ \mu m$, the representative radius of liposomes), D is the diffusion coefficient of ATP in the cytoskeleton network. The diffusion of ATP through the network of cytoskeletal filaments can be predicted as a function of the excluded volume fraction of actin by percolation theory [7,8]:

$$D_{ATP} = D_0 \left(1 - \frac{\phi_{ex}}{\phi_c}\right)^{\mu}$$

where $\phi_c = 0.942$ and $\mu = 1.6$ are two fitting parameters [9]. D_0 is diffusion of ATP in the solution without actin filaments. The excluded volume of actin (ϕ_{ex}) can be measured from the porosity of the actin network (Supplemental Figure 11) as $\phi_{ex} = 1 - \phi$. In our experiments with VCA, $\phi_{ex} = 0.8 \sim 0.95$. Choose D_0 as the

diffusion of ATP in $50mg/mL$ dextran solution, which is about twice than that in the physiological solutions [10], therefore, $D_0 = 7.5 \times 10^{-11} m^2/s$ [11], we obtain $D_{ATP(\phi_{ex}=0.7)} = 8.5 \times 10^{-12} m^2/s$, $D_{ATP(\phi_{ex}=0.8)} = 3.6 \times 10^{-12} m^2/s$, $D_{ATP(\phi_{ex}=0.9)} = 5.17 \times 10^{-13} m^2/s$. They agree with the previously published results for the diffusion of ATP in the actin network [12-14].

If $\phi_{Th} > 1$, this scenario is diffusion-limited, our assumption above is verified. We can also calculate the Thiele length defined as:

$$L_{Th} = \frac{r}{\phi_{Th}}$$

The Thiele modulus and the Thiele length with different choice of the diffusion coefficient of ATP (D_{ATP}), and the first-order reaction rate (k_1) are shown in Supplementary Figure 12.

Supplementary Note 3: Active gel model for pore formation and expansion in Actomyosin Liposomes

Physical description of the actomyosin liposome. Here we study the stability and formation of a single pore within a thin actomyosin liposome, adherent to a substrate underneath. The flat adherent liposome consists of two faces, the lower one is adhered to the substrate, while the pore nucleates and expands in the upper face. The adhesion stress occurs at the lower liposome surface. At very low adhesion, spontaneously formed pores are unstable and closes very fast. However, above a critical adhesion force, f^*_{adh}, the spontaneously formed pores become stable for pore size larger than a critical size R_c. At significantly high adhesion the critical pore size becomes small, $R_c \to 0$, such that the pore size grows in time to eventually rupture the liposome.

We consider a circular pore of radius $R(t)$ formed within an actomyosin liposome, with F-actin density given by $\rho(x, t)$. Mass conservation of F-actin implies:

$$\frac{\partial \rho}{\partial t} + \nabla \cdot (\rho \boldsymbol{v}) = -\frac{1}{\tau_a}(\rho - \rho_0) \qquad (1)$$

where $\boldsymbol{v}$ is the F-actin flow field, τ_a^{-1} is F-actin turnover rate, and ρ_0 is the equilibrium F-actin density. Mechanical force balance is given by:

$$\nabla \cdot [\sigma_{viscous} + \sigma_a(\rho)\boldsymbol{I} - P(\rho)\boldsymbol{I}] = \Gamma \boldsymbol{v} - \boldsymbol{f}_{adh} \qquad (2)$$

where P is the osmotic pressure difference, σ_a is the active stress, $\sigma_{viscous}$ is the viscous stress, Γ is the friction coefficient, and $\boldsymbol{f}_{adh}$ is the adhesive force density originating from the substrate adhesion.

The system has two modes of dissipation namely viscous dissipation in the cortex-membrane fluid layer and the frictional dissipation due to the movement of this outer cortex-membrane layer relative to the internal fluid in liposome bulk. Due to the small magnitude of the velocity of pore opening ($v \sim 10^{-3}$μms^{-1}) and relatively high substrate adhesion in experiments, we do not consider the effect of friction in the formation and expansion of the liposome pore. In that approximation, the force balance equation becomes

$$\nabla \cdot \sigma_{tot} = -\boldsymbol{f}_{adh} \tag{3}$$

where σ_{tot} is the total internal stress in the gel. However, to investigate the spatial patterns of actomyosin flow during pore expansion we take both the viscous and frictional dissipation mechanisms into account as their competition gives rise to an important length scale dictating the spatial localization of F-actin flow field.

The pore opening process involves the competition between the effective internal contractile stress that acts to open the pore, and the membrane tension γ that closes the pore. In the given experimental setup, the liposome has ~0 membrane stress (as there is enough excess membrane) prior to adhesion. But upon adhesion membrane stress increases as adhesion stretches the liposome. Above a critical value this increasing adhesive force builds up enough membrane stress which leads to pore expansion and liposome rupture.

Dynamics of pore expansion. The pore expansion does not occur during the first stage of liposome-substrate adhesion but happens in a later stage of increased adhesion force. We assume a constant adhesive force $\boldsymbol{f}_{adh}$ during pore opening as the adhesive force build up time (~ 20 min) is much longer than the timescale of pore opening (~ 20 - 100 s). To derive the dynamical equation for pore radius *R*(*t*), we assume rotational symmetry and write the force-balance equation in polar coordinates as,

$$\frac{d\sigma_{rr}}{dr} + \frac{\sigma_{rr} - \sigma_{\theta\theta}}{r} = -f_{adh} \tag{4}$$

where,

$$\sigma_{rr} = \eta \frac{dv_r}{dr} + \sigma_a(\rho) - P(\rho) \tag{5}$$

$$\sigma_{\theta\theta} = \eta \frac{v_r}{r} + \sigma_a(\rho) - P(\rho) \tag{6}$$

The force balance equation is then given by:

$$\eta \frac{d^2 v_r}{dr^2} + \frac{\eta}{r}\left(\frac{dv_r}{dr} - \frac{v_r}{r}\right) + \frac{d}{dr}(\sigma_a - P) = -f_{adh} \quad (7)$$

The continuity equation is expressed as:

$$\frac{\partial \rho}{\partial t} + \frac{d(\rho v_r)}{dr} + \frac{\rho v_r}{r} = -\frac{1}{\tau_a}(\rho - \rho_0) \quad (8)$$

Linearizing about the equilibrium, $\rho = \rho_0 + \delta\rho$, we get

$$\frac{\partial \delta\rho}{\partial t} + \rho_0\left(\frac{dv_r}{dr} + \frac{v_r}{r}\right) = -\frac{\delta\rho}{\tau_a} \quad (9)$$

Furthermore, assuming $\sigma_a(\rho) \approx \sigma_a^0 + \alpha_1\delta\rho$, and $P(\rho) \approx P_0 + \alpha_2\delta\rho$, we get,

$$\eta \frac{d^2 v_r}{dr^2} + \frac{\eta}{r}\left(\frac{dv_r}{dr} - \frac{v_r}{r}\right) + \zeta\frac{d\delta\rho}{dr} = -f_{adh} \quad (10)$$

where $\zeta = \alpha_1 - \alpha_2$ is the measure of contractile activity. Density fluctuations relax faster than the timescale of gel deformations, $\partial_t\delta\rho = 0$. We can thus express Eq. (10) as

$$(\eta - \rho_0\tau_a\zeta)\frac{d^2 v_r}{dr^2} + \frac{(\eta - \rho_0\tau_a\zeta)}{r}\left(\frac{dv_r}{dr} - \frac{v_r}{r}\right) \approx -f_{adh} \quad (11)$$

which simplifies to:

$$\frac{d^2 v_r}{dr^2} + \frac{1}{r}\left(\frac{dv_r}{dr} - \frac{v_r}{r}\right) \approx -\frac{f_{adh}}{(\eta - \rho_0\tau_a\zeta)} \quad (12)$$

The above equation is solved for $R \leq r \leq R_L$, where R is the radius of the pore and R_L is the radius of the liposome. Using the boundary conditions, $v_r(r = R) = \dot{R}$, and $v_r(r = R_L) = 0$, the solution for v_r is given by:

$$v_r = \frac{R\dot{R}(r^2 - R_L^2)}{r(R^2 - R_L^2)} - \left(\frac{f_{adh}}{3r(\eta - \rho_0\tau_a\zeta)}\right)F_1 \quad (13)$$

Where $\dot{R} \equiv dR/dt$ is the speed of expansion of the pore radius, and $F_1 = \frac{(r-R)(r-R_L)(RR_L + r(R+R_L))}{(R+R_L)}$.

To determine the equation of motion for R we use the boundary condition, $\sigma_{rr}(r = R) = 2\gamma/R$, where γ is the membrane tension acting on the pore boundary. This gives us,

$$\dot{R} = \left(\frac{f_{adh}R(R-R_L)^2(R+2R_L)}{3(\eta R_L^2+R^2(\eta-2\rho_0\tau_a\zeta))}\right) - \left(\frac{(R^2-R_L^2)(R\sigma_0-2\gamma)}{\eta R_L^2+R^2(\eta-2\rho_0\tau_a\zeta)}\right) \quad (14)$$

where $\sigma_0 = \sigma_a^0 - P_0$ is the effective contractile stress in the liposome. We use the above equation to fit the experimental data for the dynamics of pore radius for an actomyosin liposome, with $\sigma_a^0 \neq 0$, $\tau_a \neq 0$ (Fig. 5i). In the case of F-actin liposomes, we have $\sigma_a = 0$, leading to slower expansion of the pore radius. For empty liposomes, we have $\zeta = 0$. Linearizing equation 14 and using small pore limit we get

$$\frac{dR}{dt} \approx [(\sigma_0 + 2f_{adh}R_L/3)R - 2\gamma]/\eta_{eff} \quad (15)$$

where η_{eff} is the effective viscosity of the active gel, given by $\eta_{eff} = \eta + (\eta - 2\rho_0\tau_a\zeta)R^2/R_L^2$.

Pore formation and stability. To analyze the effect of adhesion stress on pore expansion (Fig. 5h), we consider the simplest case of an empty vesicle adherent to a substrate underneath. Here we take the reasonable assumption that the effect of contractile stress σ_0 is negligible, as the liposome does not show pore expansion without adhesion. Rescaling variables as $\tilde{R} = R/R_L$ and $t = t/\tau_a$ we get the dimensionless form for the equation of pore expansion:

$$\dot{\tilde{R}} = \left(\frac{\tilde{R}-1}{(\tilde{R}^2+1)}\right)[\tilde{f}_{adh}\tilde{R}(\tilde{R}-1)(\tilde{R}+2) + 6\tilde{\gamma}(\tilde{R}+1)] \quad (16)$$

where $\tilde{f}_{adh} = f_{adh}R_L\tau_a/\eta$ and $\tilde{\gamma} = \gamma\tau_a/\eta R_l$. From a fixed point analysis of the above equation we find that pore expansion is only possible for adhesive forces higher than a critical value f_{adh}^*, given that the initial pore size is larger than a critical radius, R_c (Fig. 5h). It is also evident that for spontaneously formed small pores (due to fluctuations) to expand we need a high enough value of adhesive force, such that the pore size eventually reaches R_L, leading to liposome rupture.

It is to be noted that we do not consider the effect of "leaking" through the pore (pore size dependent stress relaxation), and we do not have any controlled way of inducing pores of different sizes (greater than critical pore size) in the experiment. With such added physical considerations and experiments our study can be extended to predict events such as stable pores without liposome rupture, and transient pore opening in the low adhesive force regime.

Dynamics of F-actin density. Pore expansion in the liposome leads to an increase in F-actin density (Fig. 5e-inset). To compute the dynamics of mean F-actin density, we use the following mass conservation equation,

$$\bar{\rho}(t)A(t) = 2\pi R_L^2 \rho_0 \quad (17)$$

where $\bar{\rho}(t)$ is the mean F-actin density, and $A(t) = 2\pi R_L^2 - \pi R(t)^2$ is the instantaneous liposome surface area. The dynamics of the mean density $\bar{\rho}/\rho_0$ is thus given by,

$$\frac{\bar{\rho}(t)}{\rho_0} = \frac{2R_L^2}{2R_L^2 - R^2(t)} \quad (18)$$

which is completely determined by the dynamics of the radius $R(t)$. We use the above equation to determine F-actin density for different compositions of the liposome (Fig. 5j).

F-actin flow during pore expansion. Experimental data (Fig. 5) show spatially localized F-actin flow patterns during pore opening. Our active gel model can be used to predict the spatiotemporal dynamics of F-actin flow. Using Eq. (2), we arrive at the equation for flow velocity:

$$\partial_r^2 v + \frac{1}{r}\left(\partial_r v - \frac{v}{r}\right) = \frac{\Gamma v - f_{adh}}{\eta_a} \quad (19)$$

Where $\eta_a = \eta - \rho_0 \tau_a \zeta$. Note that $\sqrt{\eta_a/\Gamma}$ determines the hydrodynamic length scale over which F-actin flow velocity is localized. The flow equation is subject to the boundary conditions $v(r = R) = \dot{R}$ and $v(r = R_L) = \frac{R}{R_L}\dot{R}$. Rescaling velocity by $\bar{v} = R_L/\tau_a$ and space as $x = r/R_L$ we derive a dimensionless form for the flow profile

$$\tilde{v}'' + \frac{1}{x}\left(\tilde{v}' - \frac{\tilde{v}}{x}\right) = \tilde{\Gamma}\tilde{v} - \tilde{f}_{adh} \quad (20)$$

with $\tilde{v}' = \partial_x(v/\bar{v})$, $\tilde{\Gamma} = \Gamma R_L^2/\eta_a$ and $\tilde{f}_{adh} = f_{adh} R_L \tau_a/\eta_a$. The above equation can be analytically solved in terms of special functions, but the solution is not very informative. Instead, we numerically fit the solution to Eq.(20) to experimental data, and compute the F-actin flow kymograph as shown in Supplemental Fig. 14.

Supplementary Figures

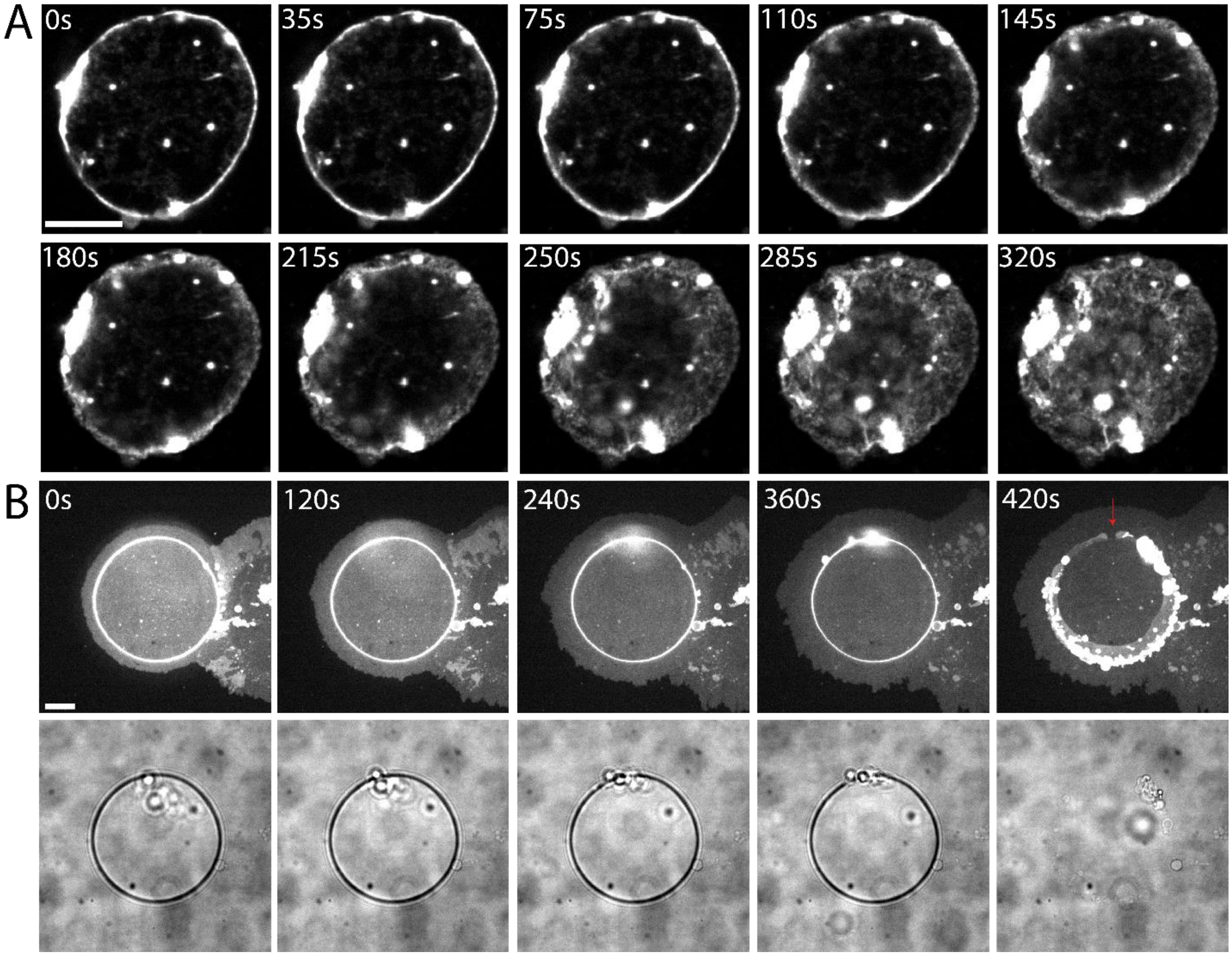

Supplemental Figure 1. Flattening of liposomes upon adhesion. (A) Actin within an actomyosin liposome imaged a confocal plane (~200 nm) above the coverslip, shows that within minutes the liposome will adopt a flattened shape, where both the basal and apical portions of the liposome are visible within the same confocal slice. The coverslip is coated with 2 mg/mL PLL. **(B)** OG-DHPE within a bare liposome shows the spreading of a liposome in a bilayer that protrudes from the base of the liposome, inducing rupture, at the red arrow. The strong spreading flattens the liposome. Coverslip is coated with 1 mg/mL PLL. Scale bars are 10 μm.

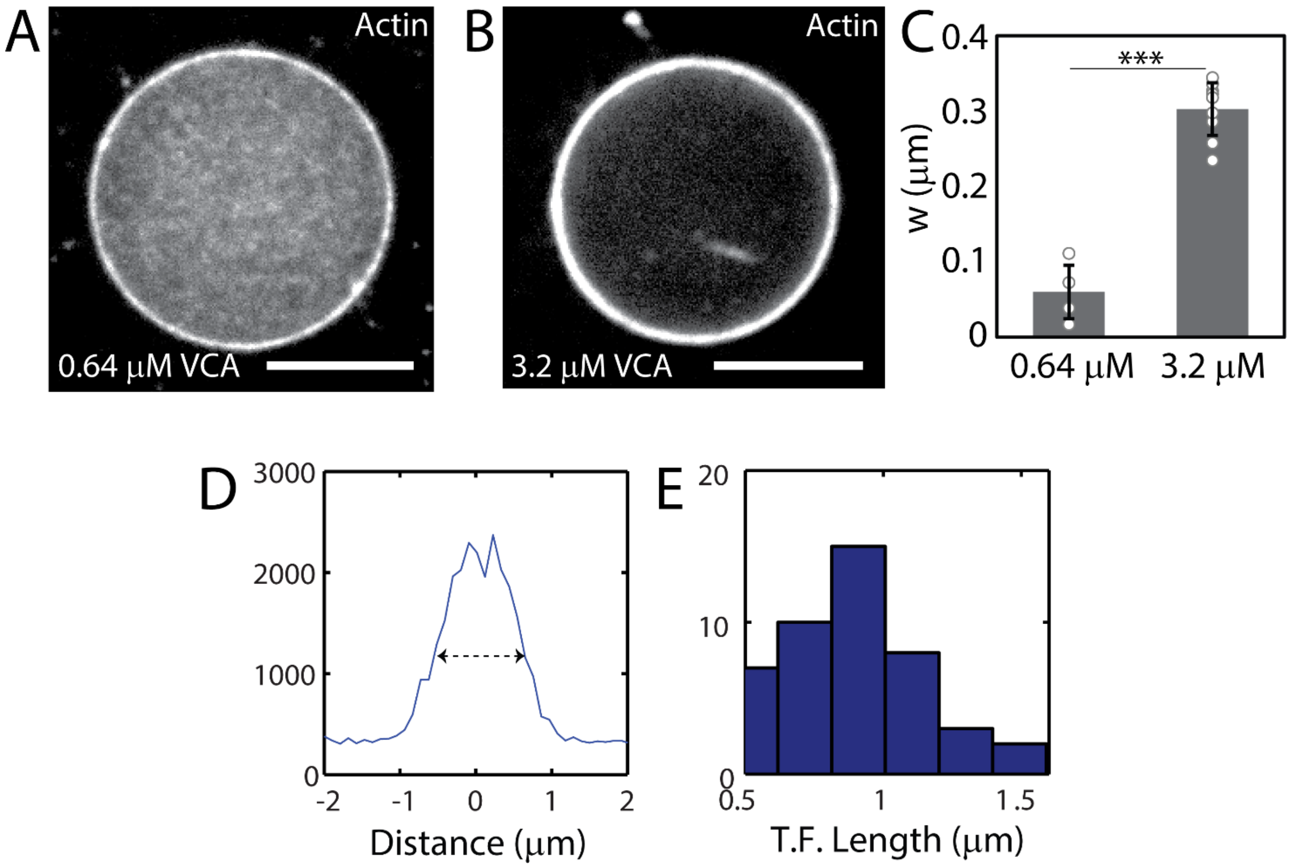

Supplemental Figure 2. Components of actin and actomyosin liposome: 0.64 μM VCA **(A)** & 3.2 μM VCA **(B)** within the liposome determines the thickness of the cortical shell **(C)**. N_{low}=4, N_{high}=9. **(D)** Lines scan of a single myosin thick filament. Arrow indicates half-maximum region, over which the length of the thick filament is estimated. **(E)** Distribution of filament lengths. N_{tot}=45. Scale bars are 10 μm. *** indicates p<0.001.

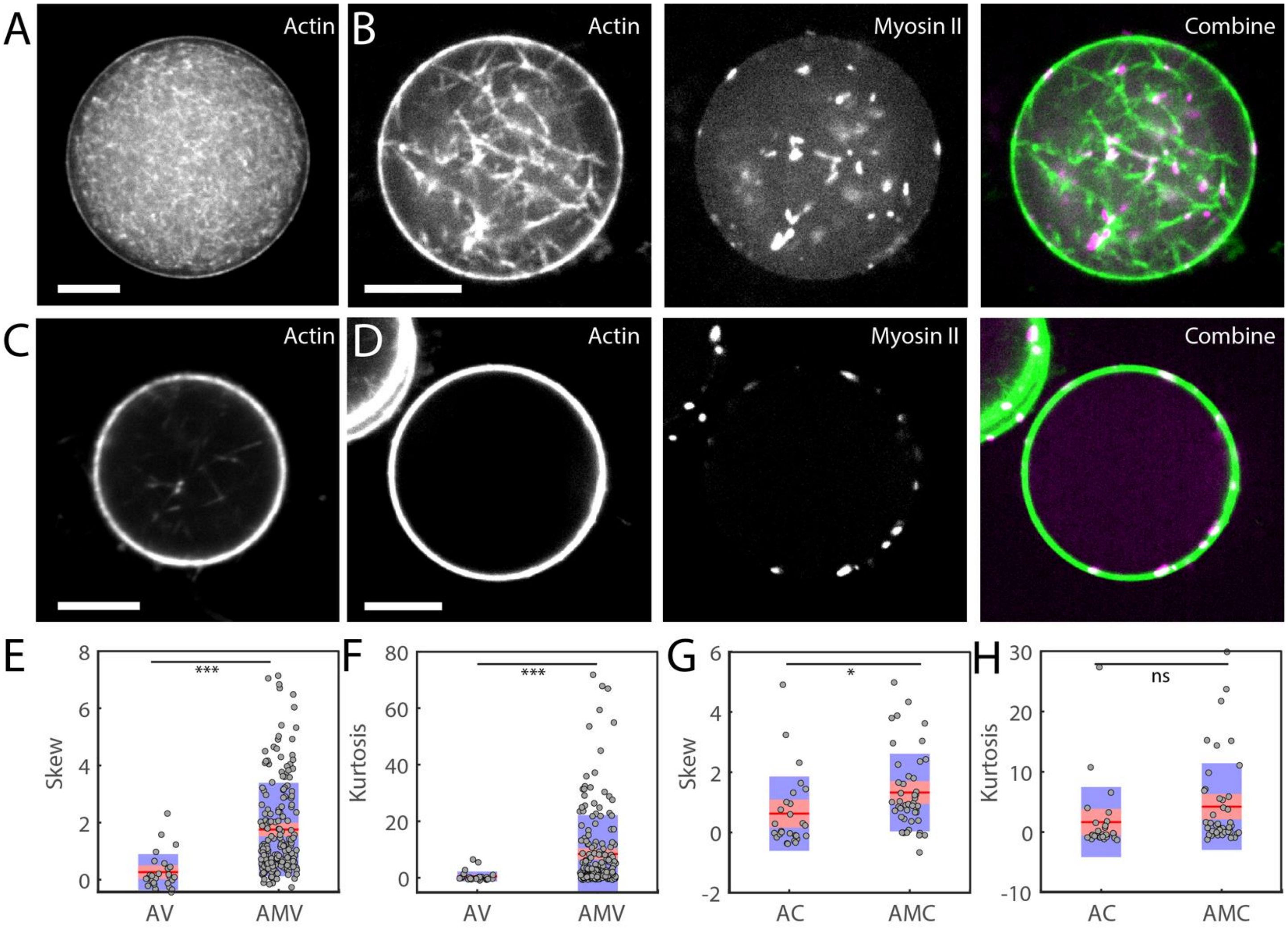

Supplemental Figure 3. **Myosin alters the organization of F-actin within liposomes.** **(A)** Actin within a liposome, without myosin (AV). **(B)** Actin and full length myosin within a liposome (AMV). **(C)** Actin nucleated by Arp2/3, in the presence of gelsolin and cofilin (in the absence of myosin) generates a layer, or "cortex" (AC). **(D)** Actin cortex, in the presence of myosin. **(E)** Skew and **(F)** kurtosis of the distribution of actin fluoresence within 2D image of actin for AV & AMV. N_{av}=27, N_{amv}=182 (Total), N_{av}=2, N_{amv}=3 (Independent Experiments). **(G)** Skew and **(H)** Kurtosis for the distribution of fluoresence along the circumference of the actin ring. N_{ac}=27, N_{amc}=45 (Total), N_{ac}=4, N_{amc}=3 (Independent Experiments). Scale bars are 10 µm. *** indicates $p<0.001$. * indicates $p = 0.05$. ns is non-significant.

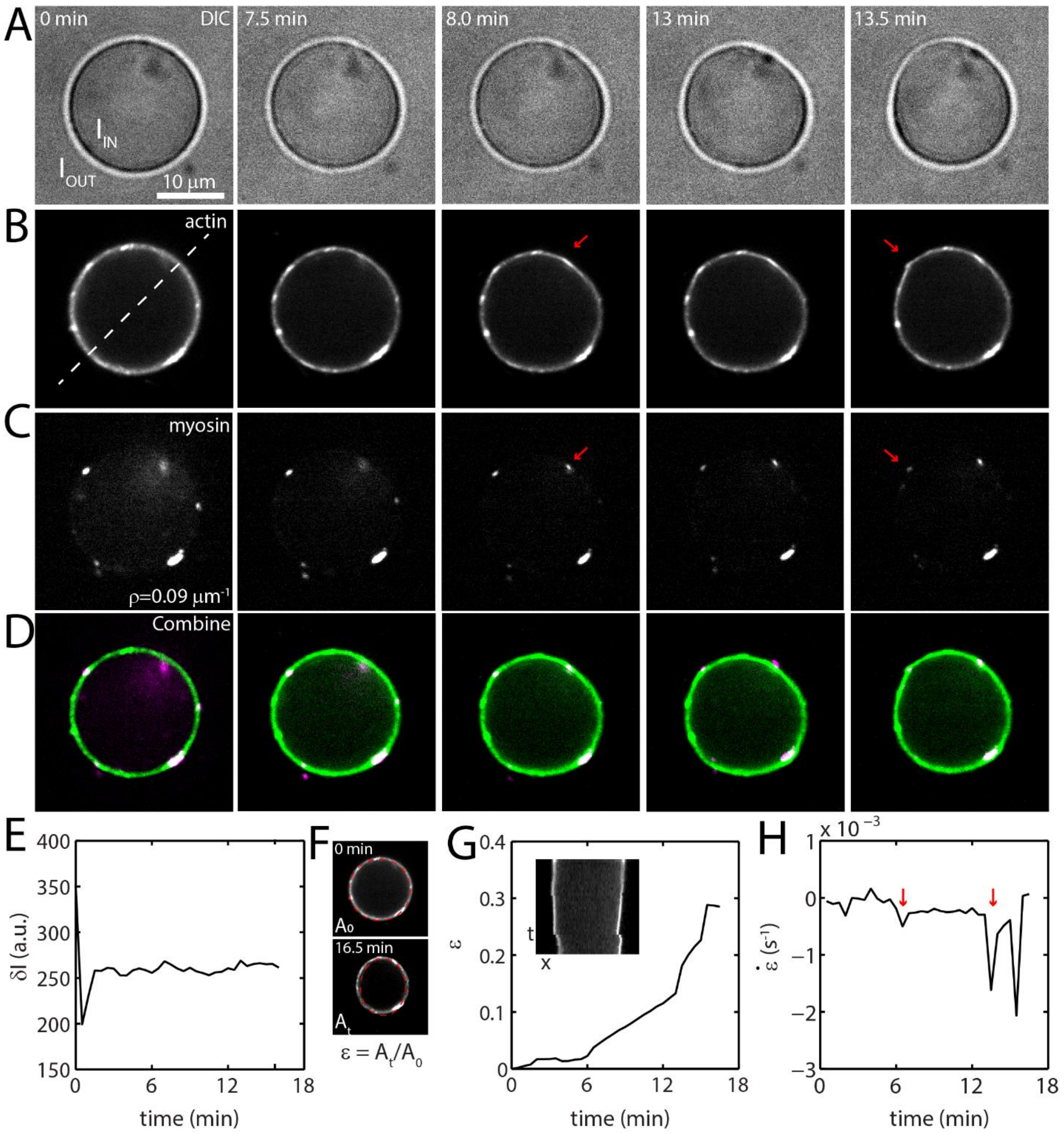

Supplemental Figure 4. Shape transformations in actomyosin liposomes at low myosin density. Phase **(A)**, actin **(B)**, myosin **(C)** and actin & myosin combine **(D)** of an active and contractile non-adherent liposome. Red arrows show contractile events in proximity to myosin thick filaments. **(E)** Change in light intensity ($\delta I = I_{out}$-I_{in}) within the liposome in A. The change in fluorescence reflects spontaneous changes in permeability and access to ATP in the external solution. **(F)** Indication of strain measurement. **(G)** Strain in liposome, ε over time. **(G, inset)** Kymograph through the F-actin in liposome over time. **(H)** Strain rate, $\dot{\varepsilon}$ over time. Red arrows indicate the shape changes that can be seen in parts A through D.

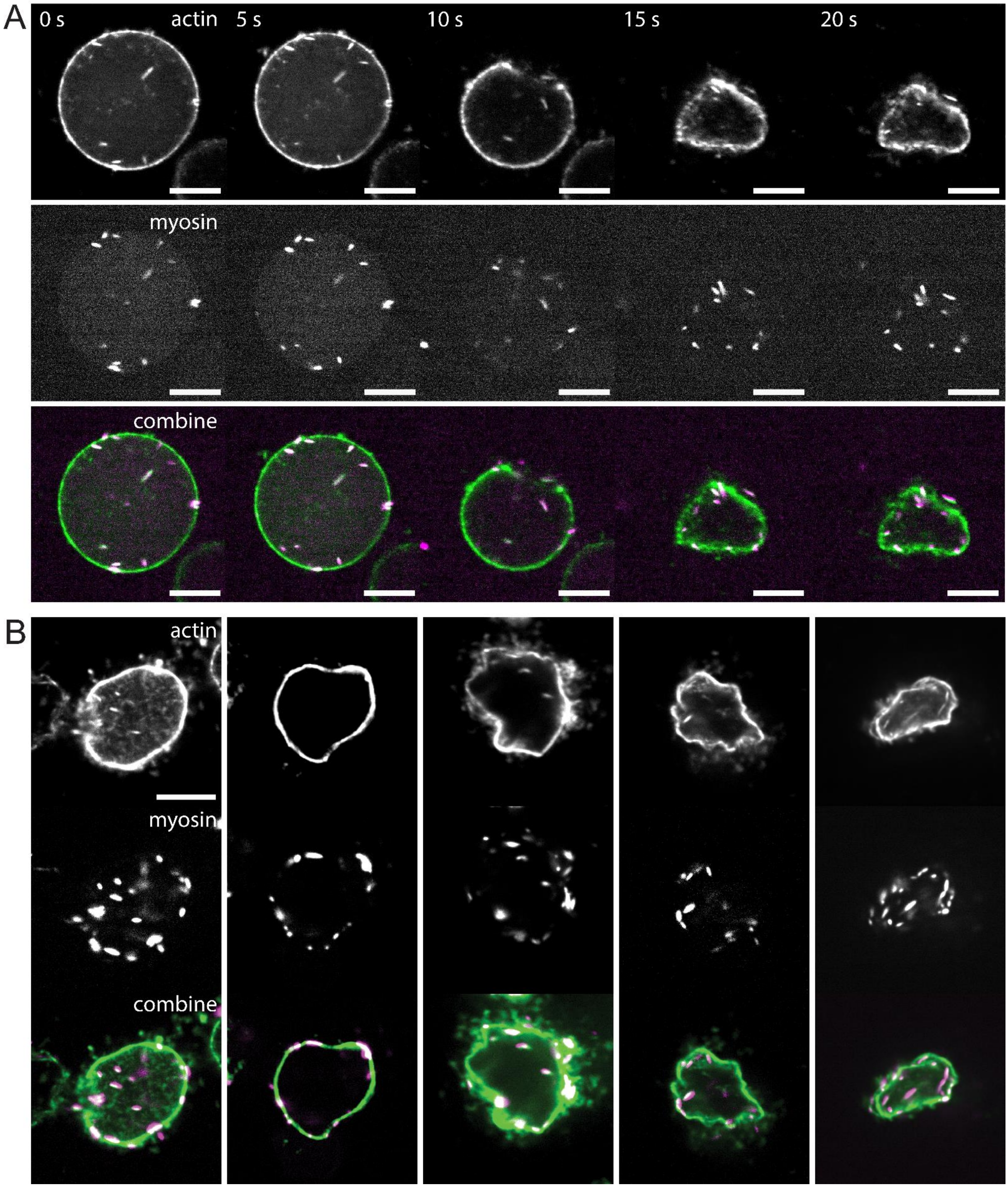

Supplemental Figure 5. Shape transformation in actomyosin liposomes at high myosin concentration. The dynamics (**A**) and end points (**B**) of actin, myosin, and a color combine for high-density myosin liposomes that have ruptured the membrane and contracted. Scale bar is 10 µm.

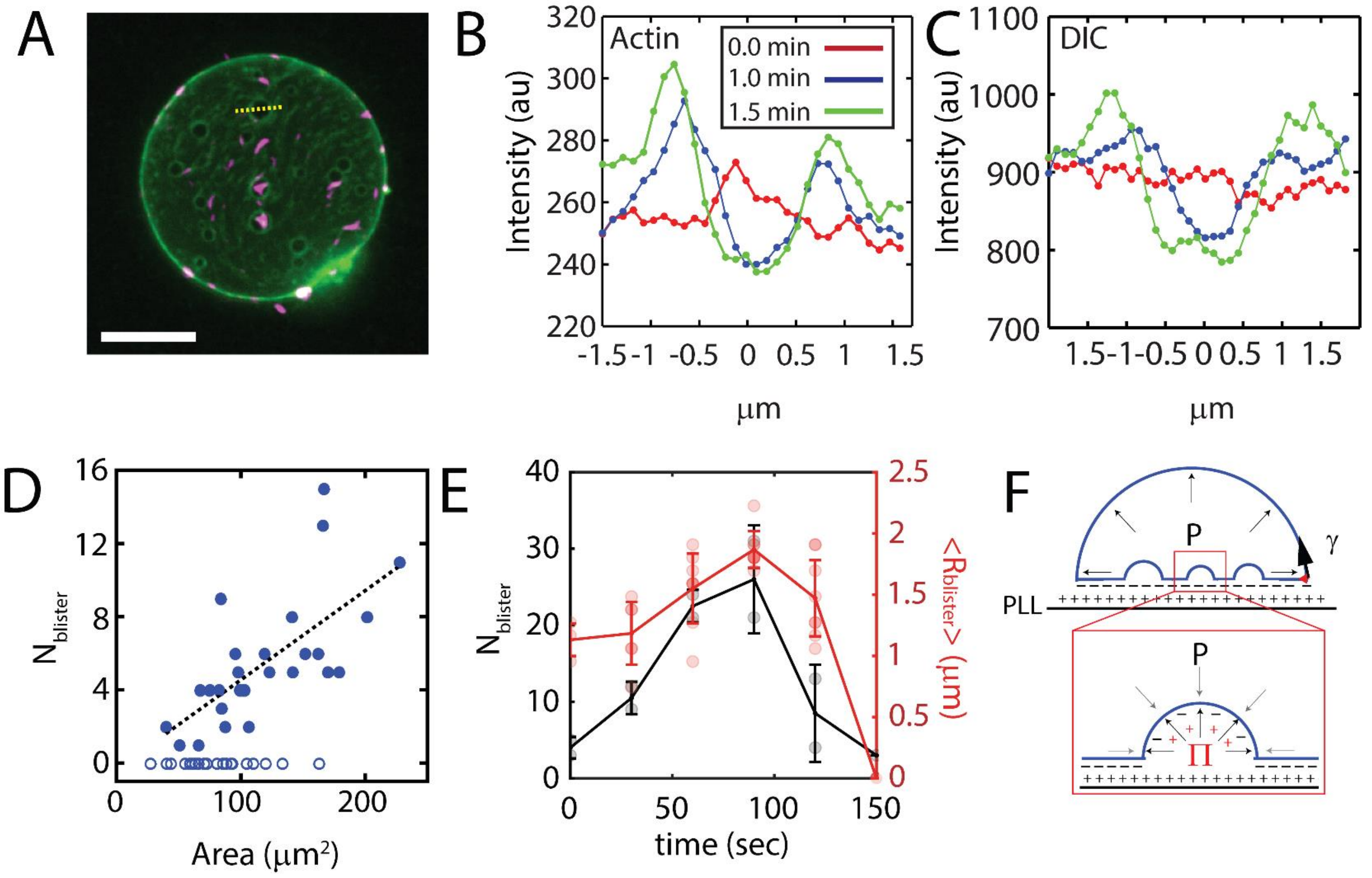

Supplemental Figure 6. Blisters reflect accumulation of internal mechanical stresses. (A) Actomyosin liposome adhered to PLL coated coverslip exhibiting blisters (dark areas in actin signal). Actin is in green, myosin in magenta. Yellow dotted line is used for linescans in B and C. Scale bar is 10 μm. **(B)** Linescan over actin channel, which shows a decrease in actin in the center, and an increase on the sides. **(C)** Linescan over DIC channel, also showing change in intensity. **(D)** The number of blisters increases with the size of the liposome (solid circles) although not all liposomes have blisters (open circles). **(E)** Number and size of blisters over time. Early in P3, blisters are formed, but disappear immediately prior to rupture. **(F)** Schematic representation of blisters. Charge is sequestered at the basal surface of the liposome, creating an osmotic pressure. The osmotic pressure pushes upward into the liposome, displacing the membrane and actin layer. Over time, membrane tension and the internal pressure increase, and decrease the size and number of blisters.

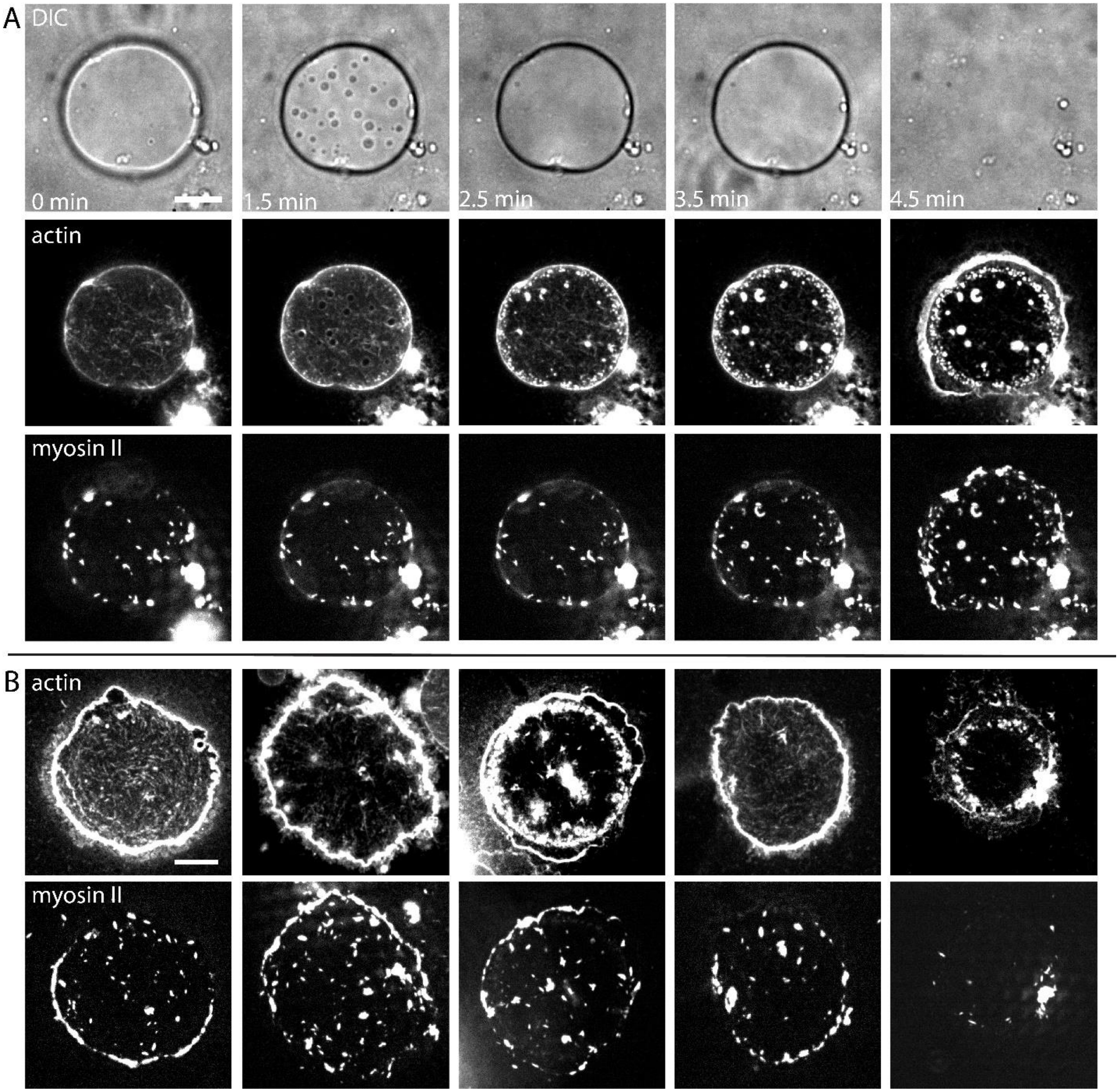

Supplemental Figure 7. Actin distributions in liposomes that have adhered and ruptured. **(A)** DIC, actin, and myosin images of a liposome adhered to a coverslip coated in 2 mg/mL PLL. Rupture shows a dramatic increase in F-actin fluorescence. Scale bar is 10 µm. Time is from first observation of the liposome. **(B)** Actin and myosin II images, of actomyosin liposomes that have adhered and ruptured on 2 mg/mL PLL-coated coverslips. Images show differences in the number and distribution of F-actin "spots" and "clusters", shape anisotropy as well as the accumulation of actin and myosin at the border, post pore-opening. Scale bars are 10 µm.

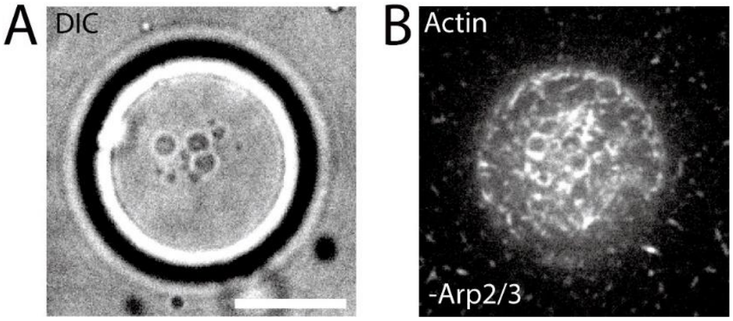

Supplemental Figure 8. Neither spots nor clusters are observed in the absence of Arp2/3. (A) Brightfield and (B) actin of blister appearance and actin growth in the absence of Arp2/3, cofilin or gelsolin. The coverslip is coated with 2 mg/mL PLL. Scale bar is 10 μm.

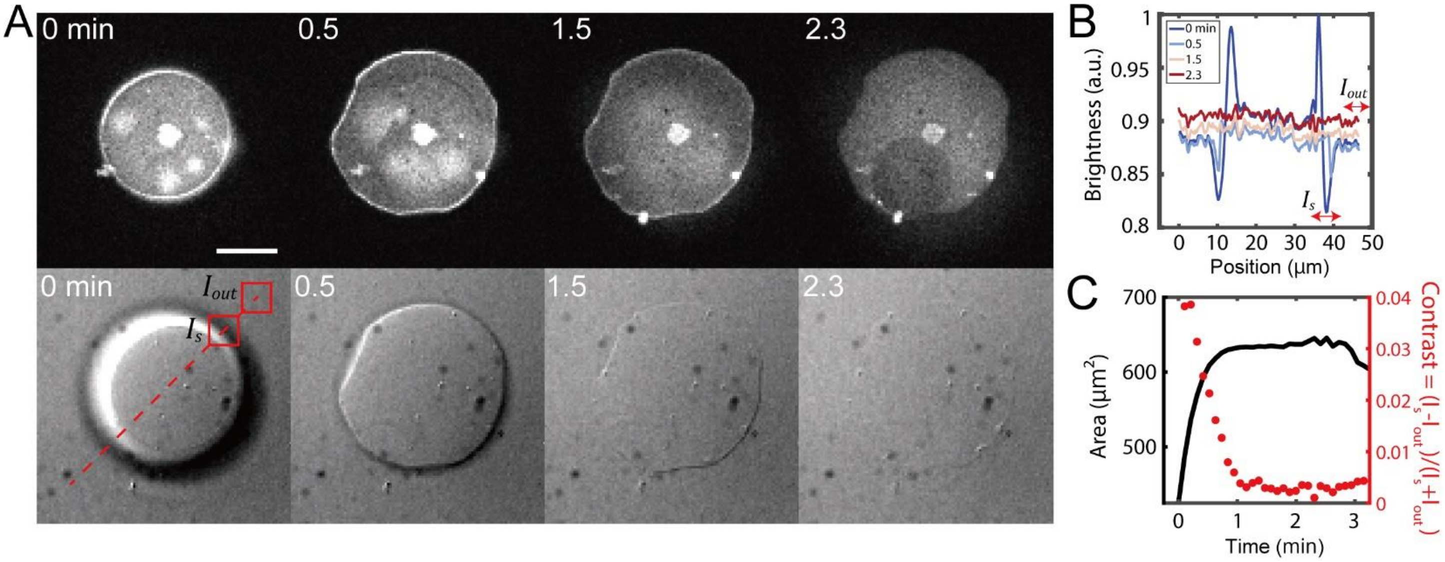

Supplemental Figure 9. Membrane is permeabilized during spreading and flattening of a bare liposome. (A) Fluorescent DHPE of a bare liposome adhered to 10 mg/ml PLL on a glass (top) and DIC (bottom). (**B**) Brightness is extracted from the red broken line in DIC in A. Brightness around the surface of the liposome is defined as I_s = min(Brightness) at 38 < Position < 41 μm, while the brightness outside of the liposome is defined as I_{out} = mean(Brightness) at 41 < Position < 43. **(C)** Adhesion area is calculated from DHPE in A. Contrast is defined as Contrast = $|I_s - I_{out}|/(I_s + I_{out})$. Scale bar is 10 μm.

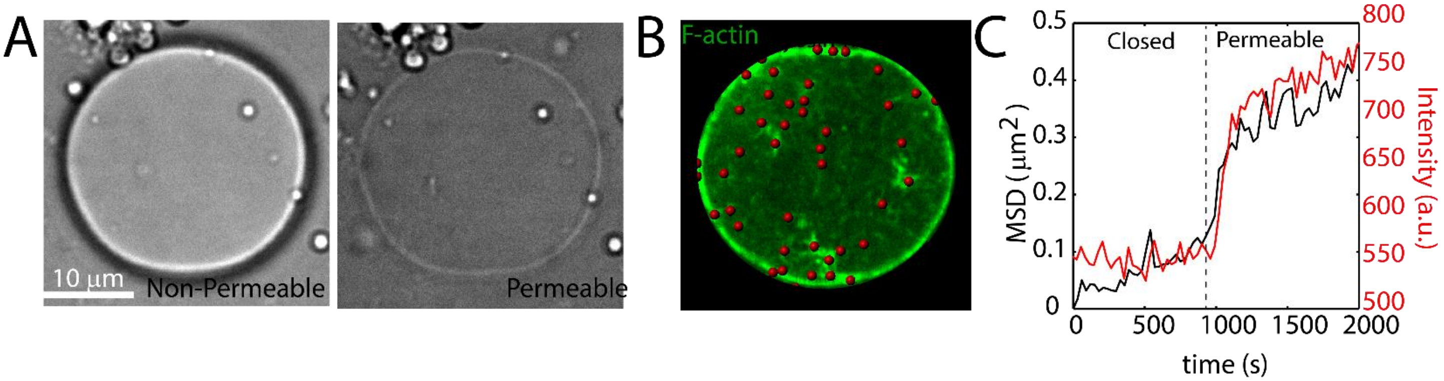

Supplemental Figure 10. Myosin motor activity is activated during permeabilization immediately before rupture. (A) DIC at early times in the P3 stage (left) and DIC at late times in P3 (right) immediately before rupture (P4). **(B)** F-actin (green) and spot tracked myosin thick filaments (red dots). **(C)** Mean-Squared Displacement (MSD) of myosin motion within liposome in B (Supplementary Note 1). Scale bar is 10 μm.

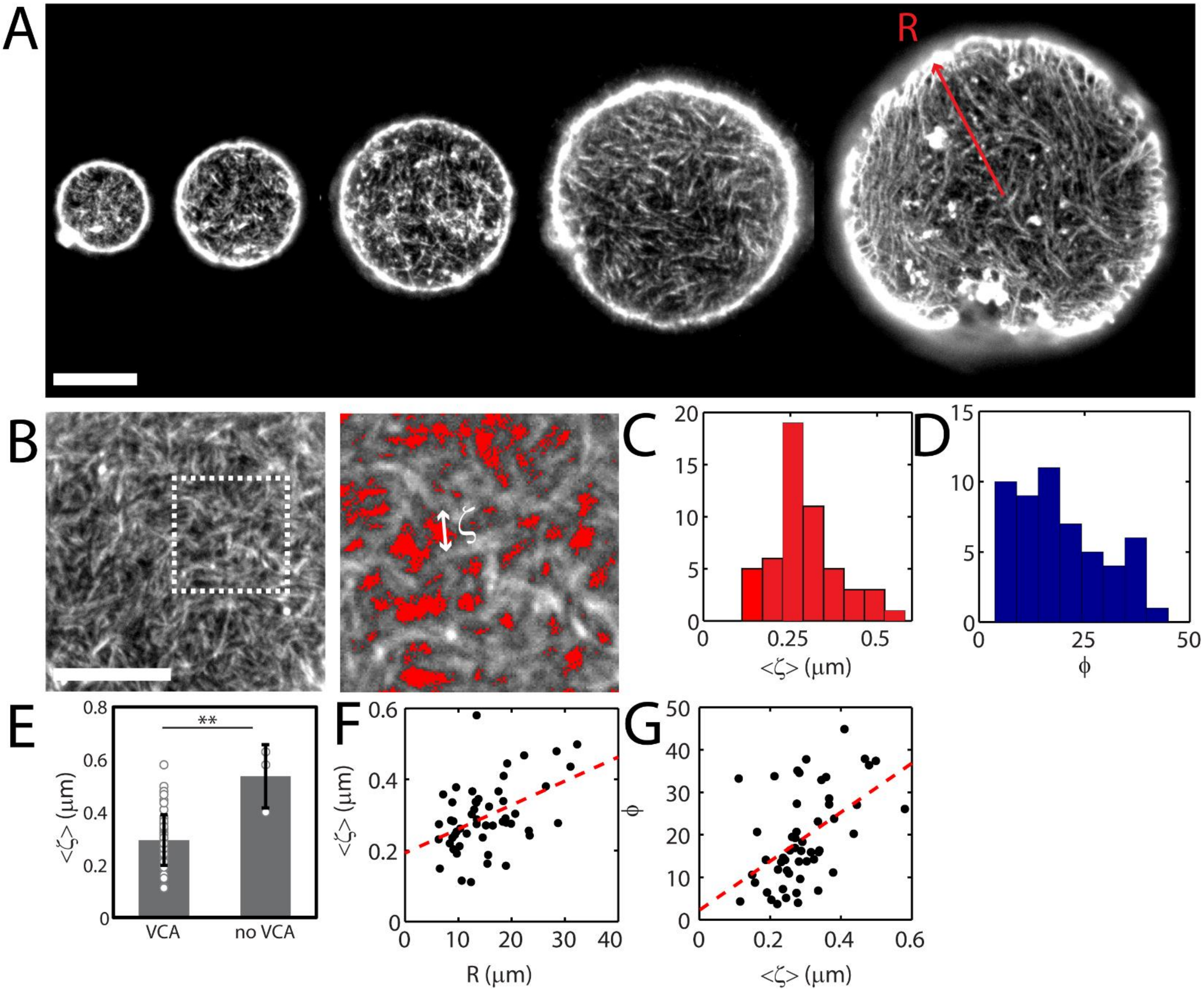

Supplemental Figure 11. Poroelastic Parameters. (**A**) F-actin within liposomes adhered to poly-histidine covered coverslips. Scale bar is 10 μm. Red line indicates the radius of the liposome, *R*. (**B**) Zoom-in on F-actin within an adherent liposome. Dotted square indicates region for zoom-in. Red areas indicate pores, with size ζ. Scale bar is 2 μm (**C**) Average pore size, $\langle\zeta\rangle$. N=53 actin liposomes. (**D**) Average porosity, ϕ. (**E**) Mean pore size for Arp2/3 nucleated F-actin, and F-actin polymerized in the bulk of the liposome. N=53 for VCA and N=3 for no VCA. (**F**) Mean pore size as a function of radius size, *R*. (**G**) Relationship between porosity and pore size.

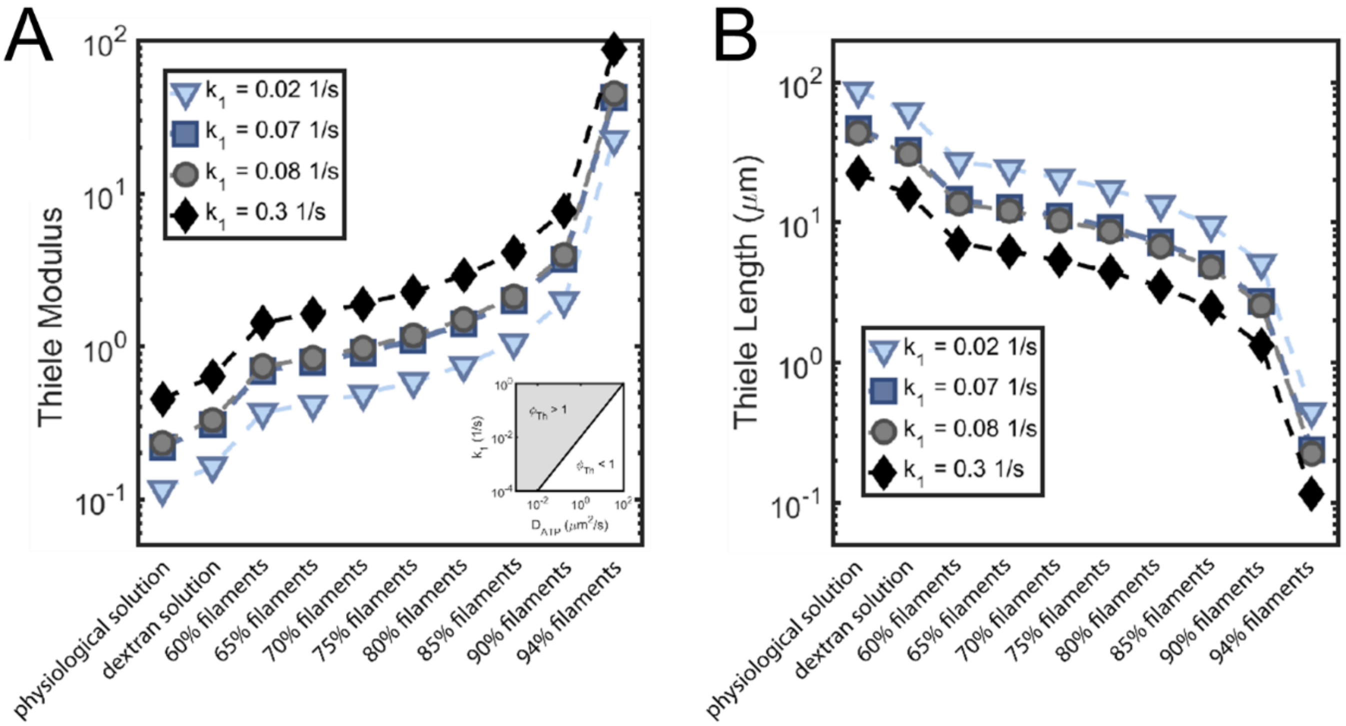

Supplemental Figure 12. Thiele modulus and Thiele length. (**A**) Relationship between Thiele modulus and different porosity conditions. (**B**) Relationship between Thiele length and different porosity conditions.

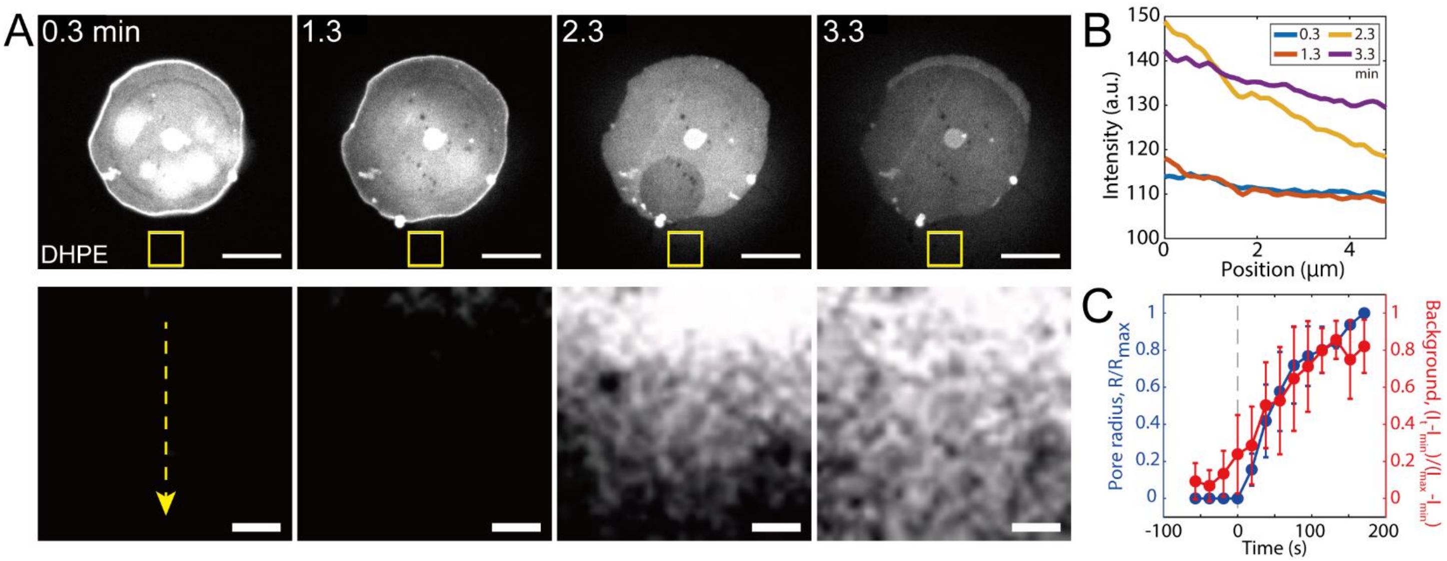

Supplemental Figure 13. Leakage of lipids during pore opening. (A) Fluorescent DHPE of a bare liposome adhered to 10 mg/ml PLL on a glass (top). Scale bar is 10 µm. Yellow square indicates the region in which the background intensity is extracted (bottom, zoom-in). Scale bar is 1 µm. (**B**) Intensity profile is extracted along the broken arrow in A. **(C)** Pore radius (left, in blue) and the background intensity (right, in red) over time (N=6, n=6 independent experiments). Time at 0 s corresponds to the onset of the pore opening. Increase in the background intensity shows the leakage of lipids from the ruptured liposome to the bottom glass substrate. Because the density ρ is calculated from the mass conservation Eq. (17), this assumption is no longer applicable in the presence of the leakage of lipids (Fig. 5J).

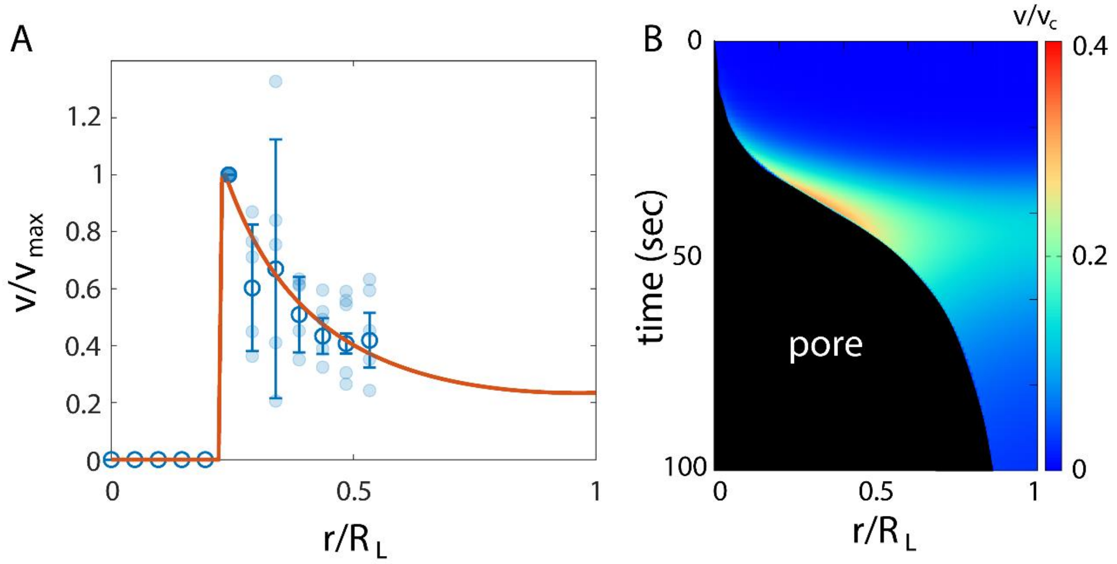

Supplemental Figure 14. Pore Opening. (A) Spatial profile of actin flow calculated by Particle Image Velocimetry during pore opening, radially averaged around the pore. Solid line is a fit of the active gel model to experiments. Flow is localized around the pore and decays near the boundary of the liposome. A non-zero boundary flow allows the membrane-actin layer to flow into the adherent lower surface of liposome. **(B)** A kymograph of flow velocity shows that actin flow is small during the initial period of slow pore opening, becomes significant as the pore expands rapidly, and finally reduces when the pore is very large before rupture. Here v_c is a characteristic velocity defined by the ratio of adhesive force to the friction coefficient. For parameter values see Supplementary Table 1.

Supplementary Table 1: List of variables used in experiments and simulations

Symbol	Appears in	Interpretation
$N_{blister}$	Fig. 3E	Total number of blisters observed in a liposome.
$\partial U/\partial x$	Fig. 5D	Spatial displacement gradient
ρ	Fig. 5E	Intensity of actin per unit area. ρ is instantaneous intensity density whereas ρ_0 is density at t = 0.
τ	Fig. 5F	Timescale extracted by fitting pore opening data.
η	Fig 5G	Viscosity of lipid membrane + actomyosin cortex composite. In absence of actomyosin or actin cortex, this variable represents viscosity of lipid membrane only.
γ	Fig. 5G	Surface tension of lipid membrane + actomyosin cortex composite. In absence of actomyosin or actin cortex, this variable represents surface tension of lipid membrane only.
R	Fig. 5G	Radius of pore
R_L	Fig. 5G	Radius of liposome
f_{adh}	Fig. 5H	Force of adhesion
τ_a	Fig. 5H	Actin turnover time scale
R_c	Fig. 5H	Critical radius of pore. Pores smaller than R_c are unstable and closes immediately.

	Adhesion Force f_{adh} (pN/µm^3)	Membrane tension γ (pN/µm)	Liposome Size R_L (µm)	Active Stress σ_a (Pa or pN/µm^2)	Viscosity (Pa.sec)	Friction $\tilde{\Gamma}$ (Pa.sec/µm^2)	Contractility parameter ζ
Fig 5H	Varied	0.5	20	0	1	-	-
Fig 5I-J: *Empty*	0.1	0.1	12	0	7	-	0
F-actin	0.1	0.1	12	0	15	-	-0.1
Actomyosin	0.1	0.1	12	1.05 (fitting)	15	-	0.1
SFig 14	0.25	10^{-4}	35	0	40	0.1	0.25

Actin density is taken to be 2 µM.

Supplementary Table 2: Sample Statistics

	Non-Adherent AML	Non-Adherent AL	Adherent AML	Adherent BL	Adherent AL	Num Spots	Num AL Blisters	Num AML Blisters	Num Myo TF
Fig 1	0	0	0	262	0	0	0	0	0
Fig 2	1	0	3	0	0	0	0	0	0
Fig 3A,B	0	0	2	0	0	0	0	0	0
Fig 3C,D,H	0	0	0	0	110	0	110	0	0
Fig 3E-G,I	0	0	2	0	0	0	0	12	0
Fig 4A,F	0	0	1	0	0	0	0	0	0
Fig 4B	0	163	0	0	179	0	0	0	0
Fig 4C,D	0	0	6	0	0	24	0	0	0
Fig 4E	0	0	0	0	202	0	0	0	0
Fig 4G	0	0	6	0	0	0	0	0	0
Fig 4H	0	0	1	0	0	72	0	0	0
Fig 5A-E	0	0	1	0	0	0	0	0	0
Fig 5F-J	0	0	8	12	14	0	0	0	0
SFig 1	0	0	1	1	0	0	0	0	0
SFig 2	1	13	0	0	0	0	0	0	45
SFig 3	227	54	0	0	0	0	0	0	0
SFig 4	1	0	0	0	0	0	0	0	0
SFig 5	6	0	0	0	0	0	0	0	0
SFig 6	0	0	50	0	0	0	0	148	0
SFig 7	0	0	6	0	0	0	0	0	0
SFig 8	0	0	0	0	1	0	0	0	0
SFig 9	0	0	0	1	0	0	0	0	0
SFig 10	0	0	1	0	0	0	0	0	0
SFig 11	0	0	0	0	56	0	0	0	0
SFig 12	0	0	0	0	0	0	0	0	0
SFig 13	0	0	0	6	0	0	0	0	0
SFig 14	0	0	1	0	0	0	0	0	0
Total	236	230	89	546	562	143	110	160	45
Tot Indep	14	33	24	213	27	7	5	2	1

Supplementary References

1 Froment, G. F., Bischoff, K. B. & De Wilde, J. *Chemical reactor analysis and design*. Vol. 2 (Wiley New York, 1990).

2 Pollard, T. D. & Weeds, A. G. The rate constant for ATP hydrolysis by polymerized actin. *FEBS letters* **170**, 94-98 (1984).

3 Korn, E. D., Carlier, M.-F. & Pantaloni, D. Actin polymerization and ATP hydrolysis. *Science* **238**, 638-644 (1987).

4 Pieper, U. & Wegner, A. The End of a Polymerizing Actin Filament Contains Numerous ATP− Subunit Segments That Are Disconnected by ADP− Subunits Resulting from ATP Hydrolysis. *Biochemistry* **35**, 4396-4402 (1996).

5 Carlier, M., Pantaloni, D. & Korn, E. The effects of Mg2+ at the high-affinity and low-affinity sites on the polymerization of actin and associated ATP hydrolysis. *Journal of Biological Chemistry* **261**, 10785-10792 (1986).

6 Blanchoin, L. & Pollard, T. D. Hydrolysis of ATP by polymerized actin depends on the bound divalent cation but not profilin. *Biochemistry* **41**, 597-602 (2002).

7 Halperin, B. I., Feng, S. & Sen, P. N. Differences between lattice and continuum percolation transport exponents. *Physical review letters* **54**, 2391 (1985).

8 Elam, W., Kerstein, A. & Rehr, J. Critical properties of the void percolation problem for spheres. *Physical review letters* **52**, 1516 (1984).

9 Novak, I. L., Kraikivski, P. & Slepchenko, B. M. Diffusion in cytoplasm: effects of excluded volume due to internal membranes and cytoskeletal structures. *Biophysical journal* **97**, 758-767 (2009).

10 Kushmerick, M. & Podolsky, R. Ionic mobility in muscle cells. *Science* **166**, 1297-1298 (1969).

11 Gonzalez-Castillo, C., Rubio, R. & Zenteno-Savin, T. Coronary flow-induced inotropism is modulated by binding of dextrans to the endothelial luminal surface. *American Journal of Physiology-Heart and Circulatory Physiology* **284**, H1348-H1357 (2003).

12 Alekseev, A. E. *et al.* Restrictions in ATP diffusion within sarcomeres can provoke ATP-depleted zones impairing exercise capacity in chronic obstructive pulmonary disease. *Biochimica et Biophysica Acta (BBA)-General Subjects* **1860**, 2269-2278 (2016).

13 Selivanov, V. A., Krause, S., Roca, J. & Cascante, M. Modeling of spatial metabolite distributions in the cardiac sarcomere. *Biophysical journal* **92**, 3492-3500 (2007).

14 Reyes, S., Park, S., Terzic, A. & Alekseev, A. E. KATP channels process nucleotide signals in muscle thermogenic response. *Critical reviews in biochemistry and molecular biology* **45**, 506-519 (2010).
