# Supplementary material for: Membrane tension induces F-actin reorganization and flow in a biomimetic model cortex
Source: Commun Biol. 2023 Mar 27;6:325. doi: 10.1038/s42003-023-04684-7 (PMC10043271; doi:10.1038/s42003-023-04684-7)
Supplement: Supplementary file 2 — Description of Additional Supplementary Files [file 42003_2023_4684_MOESM2_ESM.pdf]

Description of Additional Supplementary Files

File name: Supplementary Data 1
Description: The source data behind the graphs in the manuscript.

File name: Supplementary Movie 1
Description: Time-lapse movie of spreading of a bare liposome (DIC and OG-DHPE) imaged a confocal plane (~200 nm) above the coverslip. The coverslip is coated with 10 mg ml-1 PLL. Scale bar is 10 µm.

File name: Supplementary Movie 2
Description: Time-lapse movie of flattening. Actin (red) and myosin (green) within an actomyosin liposome imaged a confocal plane (~200 nm) above the coverslip showing flattening where both the basal and apical portions of the liposome are visible within the same confocal slice. The coverslip is coated with 2 mg ml-1 PLL. Scale bar is 10 µm.

File name: Supplementary Movie 3
Description: Time-lapse movie of shape transformations in an actomyosin liposomes at low myosin density. Actin (red) and myosin (green) of an active and contractile nonadherent liposome are imaged. Scale bar is 10 µm.

File name: Supplementary Movie 4
Description: Time-lapse movie of shape transformations in an actomyosin liposomes at high myosin density. Actin (red) and myosin (green) of an active and contractile nonadherent liposome are imaged. Scale bar is 10 µm.

File name: Supplementary Movie 5
Description: Time-lapse movie of blister formation in DIC, actin cluster and spots formation in an actomyosin liposome. The coverslip is coated with 2 mg ml-1 PLL. Scale bar is 10 µm.

File name: Supplementary Movie 6
Description: Time-lapse movie of blister formation in DIC, actin cluster and spots formation in an actomyosin liposome. The coverslip is coated with 2 mg ml-1 PLL. Scale bar is 10 µm.

File name: Supplementary Movie 7
Description: Time-lapse movie of spreading of an actin liposome in the absence of Arp2/3, cofilin or gelsolin. The coverslip is coated with 10 mg ml-1 PLL. Scale bar is 10 µm.

File name: Supplementary Movie 8
Description: Time-lapse movie of pore opening of an actomyosin liposome. The coverslip is coated with 2 mg ml-1 PLL. Scale bar is 10 µm.

File name: Supplementary Movie 9
Description: Time-lapse movie of myosin motor activation during permeabilization immediately before rupture. Actin (red) and myosin (green) of an actomyosin liposome is imaged. The coverslip is coated with 2 mg ml-1 PLL. Scale bar is 10 µm.
